# Massive interstitial copy-neutral loss-of-heterozygosity as evidence for cancer being a disease of the DNA-damage response

**DOI:** 10.1186/s12920-015-0104-2

**Published:** 2015-07-25

**Authors:** Yogesh Kumar, Jianfeng Yang, Taobo Hu, Lei Chen, Zhi Xu, Lin Xu, Xiao-Xia Hu, Gusheng Tang, Jian-Min Wang, Yi Li, Wai-Sang Poon, Weiqing Wan, Liwei Zhang, Wai-Kin Mat, Frank W. Pun, Peggy Lee, Timothy H. Y. Cheong, Xiaofan Ding, Siu-Kin Ng, Shui-Ying Tsang, Jin-Fei Chen, Peng Zhang, Shao Li, Hong-Yang Wang, Hong Xue

**Affiliations:** Division of Life Science, Applied Genomics Centre and Centre for Statistical Science, Hong Kong University of Science and Technology, Clear Water Bay, Hong Kong; Eastern Hepatobiliary Surgery Institute, Second Military Medical University, Shanghai, China; Department of Oncology, Nanjing First Hospital, and Collaborative Innovation Center for Cancer Personalized Medicine, Nanjing Medical University, Nanjing, China; Jiangsu Key Laboratory of Cancer Molecular Biology and Translational Medicine, Jiangsu Cancer Hospital, Nanjing, China; Department of Hematology, Changhai Hospital, Second Military Medical University, Shanghai, China; Department of Surgery, The Chinese University of Hong Kong, Hong Kong, China; Department of Neurosurgery, Beijing Tiantan Hospital, Capital Medical University, 6 Tiantan Xili, Dongcheng District, Beijing, 100050 China; MOE Key Laboratory of Bioinformatics and Bioinformatics Division, TNLIST, and Department of Automation, Tsinghua University, Beijing, 100084 China

**Keywords:** Copy number variation, Double strand break repair, Gain-of-heterozygosity, Gene conversion, Inter-homologous recombination, Loss-of-heterozygosity

## Abstract

**Background:**

The presence of loss-of-heterozygosity (LOH) mutations in cancer cell genomes is commonly encountered. Moreover, the occurrences of LOHs in tumor suppressor genes play important roles in oncogenesis. However, because the causative mechanisms underlying LOH mutations in cancer cells yet remain to be elucidated, enquiry into the nature of these mechanisms based on a comprehensive examination of the characteristics of LOHs in multiple types of cancers has become a necessity.

**Methods:**

We performed next-generation sequencing on inter-Alu sequences of five different types of solid tumors and acute myeloid leukemias, employing the AluScan platform which entailed amplification of such sequences using multiple PCR primers based on the consensus sequences of Alu elements; as well as the whole genome sequences of a lung-to-liver metastatic cancer and a primary liver cancer. Paired-end sequencing reads were aligned to the reference human genome to identify major and minor alleles so that the partition of LOH products between homozygous-major vs. homozygous-minor alleles could be determined at single-base resolution. Strict filtering conditions were employed to avoid false positives. Measurements of LOH occurrences in copy number variation (CNV)-neutral regions were obtained through removal of CNV-associated LOHs.

**Results:**

We found: (a) average occurrence of copy-neutral LOHs amounting to 6.9 % of heterologous loci in the various cancers; (b) the mainly interstitial nature of the LOHs; and (c) preference for formation of homozygous-major over homozygous-minor, and transitional over transversional, LOHs.

**Conclusions:**

The characteristics of the cancer LOHs, observed in both AluScan and whole genome sequencings, point to the formation of LOHs through repair of double-strand breaks by interhomolog recombination, or gene conversion, as the consequence of a defective DNA-damage response, leading to a unified mechanism for generating the mutations required for oncogenesis as well as the progression of cancer cells.

**Electronic supplementary material:**

The online version of this article (doi:10.1186/s12920-015-0104-2) contains supplementary material, which is available to authorized users.

## Background

As a common feature of cancer cells, LOHs have been investigated by cytogenetics, fluorescence *in situ* hybridization, comparative genomic hybridization (CGH), array-CGH, and single nucleotide polymorphism (SNP)-based microarrays [1–4]. With the application of next-generation sequencing, analysis of LOH in cancer can further be conducted at the level of single base resolution [5, 6]. However, owing to the importance of LOHs giving rise to loss of major alleles and inactivation of tumor suppressor genes, hitherto investigations of LOHs in cancers have been focused mainly on LOHs that yield homozygous-minor genotypes. Yet a comprehensive understanding of the properties and origins of LOHs in cancers requires analysis of all types of LOHs in multiple cancers.

Accordingly, in the present study next-generation sequencing was applied to determine at single-base resolution the LOHs in the genomic sequences of various types of cancers, covering not only sequence regions that have undergone loss of heterozygosity but also single nucleotide changes where a heterozygous position has mutated to a homozygous one. Thirty tumor-control pairs of six different types of cancers including glioma (glioblastoma and astroglioma), acute myeloid leukemia, gastric adenocarcinoma, hepatocellular carcinoma, primary lung cancer (pulmonary squamous-cell carcinoma, adenocarcinoma and neuroendocrinal carcinoma), and lung-to-brain metastatic adenocarcinoma were analyzed with the AluScan platform established by our laboratory, based on the capture of ~8–25 Mb/genome of inter-Alu sequences by inter-Alu PCR amplification using multiple consensual Alu sequence-based primers for next-generation sequencing [7].

The results obtained on both LOH mutations forming homozygous-major genotypes and those forming homozygous-minor genotypes have yielded a comprehensive LOH landscape across different types of cancers that identifies the outstanding characteristics of cancer LOHs: (a) occurrence of massive percentile LOH mutations of heterozygous residues in the cancer genomes, far exceeding the percentile gain-of-heterozygosity (GOH) mutations of homozygous-major residues; (b) cancer LOHs are mainly interstitial ones indicative of gene conversion rather than segmental deletion as the major underlying mechanism for their production; and (c) cancer LOHs display preferences for the production of homozygous-major genotypes over homozygous-minor genotypes, and for transitional over transversional changes. These characteristics of cancer LOHs, determined using the AluScan platform and also confirmed by the whole-genome sequences reported for a lung-to-liver metastatic cancer [8] and a primary liver cancer [9], indicate that cancer LOHs are generated mainly by repair of double-strand breaks (DSB) through interhomolog recombination with the homologous chromosome serving as repair template. The massive scale of the interhomolog recombinations called for by the cancer LOHs suggests that a defective DNA-damage response, by weakening cell cycle checkpoints in the cancer cells, allows the entry of DSB-bearing DNA into the S-phase of the cell cycle, thereby enabling interhomolog recombination and production of the LOH and tag-along GOH mutations needed by the cells during their post-oncogenesis as well as pre-oncogenesis phases.

## Methods

### DNA samples

Participation in this study was voluntary and informed consent was obtained from each of the Han Chinese patients. Institutional Ethics Committees approvals for this study were granted by Hong Kong University of Science and Technology, Second Military Medical University of Shanghai, The First Hospital of Nanjing, Jiangsu Cancer Hospital, Chinese University of Hong Kong, and Capital Medical University of Beijing. Of the thirty patients, five were diagnosed for adenocarcinoma of the stomach; five for glioma including three with glioblastoma and two with astroglioma; five for acute myeloid leukemia; five for primary hepatocellular carcinoma; five for lung cancer including two with pulmonary squamous-cell carcinoma, two with pulmonary adenocarcinoma and one with pulmonary neuroendocrinal carcinoma; and five for lung-to-brain metastatic adenocarcinoma. Detailed medical records of the patients are given in Additional file 1: Table S2 footnotes.

DNA samples from normal white blood cells and leukemia cells were prepared using phenol-chloroform extraction; and DNA samples from normal lung tissue and solid tumors were prepared using DNAzol® Reagent from Life Technologies.

Normal white blood cells were employed as controls for the tumor samples, except for Lung 4 and Lung 5 in Table 1 where normal lung tissue served as controls. In the case of leukemias, normal white blood cells were separated from leukemia cells by Ficoll density gradient centrifugation. Sequence data on the whole genome sequences of a lung-to-liver metastatic lung adenocarcinoma and its blood cell control [8], and a primary hepatitis B positive hepatocellular carcinoma and its normal liver tissue control (case number DD59) [9], were obtained online from EBI-SRA at www.ebi.ac.uk/ena/data/view/ERP001071 and NCBI Short Reads Archive (accession number SRA076160) respectively.

**Table 1:** Summary of genotype frequencies and mutation rates for cancer samples analyzed by AluScan and by WGS^a^

Sample^b^	Sex	Total^c^ (Mb)	Genotype frequency (%)			Mutation rates^d^ (%)								MM/mm Ratio^e^
			MM	mm (×10^−2^)	Mm (×10^−2^)	R_MM_ (×10^−3^)	R_GOH-M_ (×10^−3^)	R_mm_	R_GOH-m_	R_Mm_	R_LOH_	R_Mm->MM_	R_Mm->mm_	
Gastric 1	M	16.08	99.978	0.760	1.393	0.616	0.616	1.146	1.146	22.277	21.964	17.723	4.241	4.179
Gastric 2	M	24.50	99.952	2.105	2.698	0.531	0.531	0.737	0.717	0.544	0.423	0.287	0.136	2.111
Gastric 3	M	24.84	99.961	2.040	1.903	0.221	0.221	0.138	0.138	2.813	2.390	1.523	0.867	1.756
Gastric 4	M	18.22	99.969	1.327	1.798	0.297	0.297	0.414	0.414	2.809	2.565	1.924	0.641	3.000
Gastric 5	M	16.67	99.975	0.593	1.920	0.606	0.606	0.910	0.910	3.062	2.999	2.593	0.406	6.385
Glioma 1	F	18.92	99.964	1.236	2.381	0.169	0.169	0.086	0.086	1.421	1.421	1.043	0.377	2.765
Glioma 2	M	9.84	99.960	1.707	2.310	0.468	0.468	0.298	0.298	13.154	9.239	6.863	2.376	2.889
Glioma 3	M	21.08	99.959	1.973	2.118	3.166	3.071	4.257	3.920	24.009	22.307	16.484	5.823	2.831
Glioma 4	F	8.89	99.956	2.197	2.240	7.294	7.249	0.154	0.154	4.671	4.269	2.963	1.306	2.269
Glioma 5	M	10.49	99.964	1.776	1.866	4.749	4.702	10.574	9.662	26.622	26.162	21.564	4.599	4.689
Leukemia 1	F	15.36	99.960	1.650	2.367	0.091	0.085	0.039	0.039	0.303	0.275	0.248	0.028	9.000
Leukemia 2	F	13.00	99.956	1.824	2.604	0.085	0.085	0.000	0.000	0.296	0.266	0.207	0.059	3.500
Leukemia 3	M	21.91	99.954	1.799	2.777	0.059	0.059	0.101	0.101	0.427	0.329	0.279	0.049	5.667
Leukemia 4	F	17.75	99.975	0.712	1.773	0.118	0.118	0.238	0.238	1.208	1.017	0.890	0.127	7.000
Leukemia 5	M	20.98	99.975	0.847	1.689	0.243	0.243	0.675	0.675	1.383	1.185	1.016	0.169	6.000
Liver 1	F	10.85	99.980	0.780	1.237	3.881	3.844	10.520	10.284	27.103	26.731	23.083	3.649	6.327
Liver 2	M	17.73	99.962	1.310	2.511	0.717	0.717	1.421	1.291	13.814	12.736	11.927	0.809	14.750
Liver 3	M	12.04	99.955	1.926	2.574	0.590	0.590	0.862	0.862	6.387	5.226	4.323	0.903	4.786
Liver 4	M	7.93	99.965	1.359	2.122	0.605	0.593	1.763	1.391	16.162	16.102	13.131	2.971	4.420
Liver 5	M	11.08	99.957	1.984	2.274	0.352	0.352	0.227	0.227	7.302	6.587	5.357	1.230	4.355
Lung 1	M	11.94	99.952	1.956	2.821	0.260	0.260	0.899	0.899	6.980	6.920	5.495	1.426	3.854
Lung 2	M	12.96	99.956	2.400	1.963	2.454	2.454	1.479	1.318	11.635	11.439	10.653	0.786	13.553
Lung 3	F	14.23	99.964	1.368	2.207	1.680	1.673	3.236	3.236	7.291	6.495	5.858	0.637	9.200
Lung 4	M	12.49	99.965	1.286	2.234	2.811	2.811	6.476	6.413	5.661	5.410	5.016	0.394	12.727
Lung 5	F	14.46	99.950	2.057	2.896	11.609	10.633	20.134	15.261	49.690	48.161	37.751	10.411	3.626
Lung-Brain 1	M	21.69	99.934	2.688	3.884	0.807	0.807	0.583	0.566	2.125	1.840	1.425	0.416	3.429
Lung-Brain 2	M	21.68	99.943	2.432	3.208	0.858	0.844	0.512	0.493	7.346	6.627	4.572	2.056	2.224
Lung-Brain 3	F	17.04	99.948	1.894	3.302	0.564	0.564	1.209	1.178	1.707	1.120	1.013	0.107	9.500
Lung-Brain 4	M	22.79	99.948	1.886	3.292	0.931	0.917	1.024	1.024	5.052	4.679	3.332	1.346	2.475
Lung-Brain 5	M	21.04	99.937	2.708	3.625	0.528	0.528	0.158	0.140	1.285	0.931	0.695	0.236	2.944
Leukemia Sample Av.^f, g^		17.80 ± 3.74	99.964 ± 0.010	1.366 ± 0.542	2.242 ± 0.490	0.119 ± 0.072 (0.117 ± 0.073)	0.118 ± 0.073 (0.117 ± 0.073)	0.211 ± 0.275 (0.214 ± 0.278)	0.211 ± 0.275 (0.214 ± 0.278)	0.723 ± 0.528 (0.702 ± 0.505)	0.614 ± 0.449 (0.598 ± 0.431)	0.528 ± 0.391 (0.510 ± 0.372)	0.086 ± 0.059 (0.088 ± 0.060)	6.233 ± 2.006 (6.033 ± 1.959)
Leukemia Aggregate Av.^h, g^		17.80	99.964	1.336	2.224	0.124 (0.122)	0.124 (0.122)	0.168 (0.171)	0.168 (0.171)	0.677 (0.661)	0.571 (0.559)	0.490 (0.477)	0.081 (0.082)	6.063 (5.813)
Solid Tumor Sample Av.^i, g^		15.98 ± 5.10	99.958 ± 0.011	1.750 ± 0.571	2.431 ± 0.660	1.871 ± 2.666 (1.817 ± 2.563)	1.821 ± 2.512 (1.766 ± 2.402)	2.769 ± 4.663 (2.756 ± 4.664)	2.481 ± 3.862 (2.476 ± 3.856)	10.837 ± 11.503 (10.704 ± 11.460)	10.190 ± 11.261 (10.183 ± 11.233)	8.264 ± 9.065 (8.278 ± 9.049)	1.926 ± 2.347 (1.905 ± 2.332)	5.242 ± 3.753 (5.365 ± 3.909)
Solid Tumor Aggregate Av.^j, g^		15.98	99.957	1.800	2.516	1.611 (1.572)	1.565 (1.525)	2.244 (2.302)	1.972 (2.024)	8.757 (8.727)	8.160 (8.198)	6.511 (6.564)	1.650 (1.635)	3.947 (4.015)
Glioma Sample Av.^k, g^		13.84 ± 5.70	99.961 ± 0.003	1.778 ± 0.358	2.183 ± 0.202	3.169 ± 2.992 (2.928 ± 2.568)	3.132 ± 2.970 (2.885 ± 2.537)	3.074 ± 4.550 (3.065 ± 4.570)	2.824 ± 4.152 (2.808 ± 4.159)	13.975 ± 11.241 (13.430 ± 11.333)	12.680 ± 10.998 (12.792 ± 10.936)	9.783 ± 8.876 (9.888 ± 8.823)	2.896 ± 2.270 (2.904 ± 2.265)	3.089 ± 0.928 (3.157 ± 0.887)
Glioma Aggregate Av.^l, g^		13.84	99.961	1.733	2.195	2.733 (2.500)	2.691 (2.453)	3.202 (3.445)	2.943 (3.158)	13.488 (13.513)	12.290 (12.786)	9.348 (9.758)	2.943 (3.027)	3.177 (3.224)
All Sample Av.^m, g^		16.28 ± 4.89	99.959 ± 0.011	1.686 ± 0.576	2.400 ± 0.631	1.579 ± 2.515 (1.534 ± 2.419)	1.537 ± 2.374 (1.491 ± 2.273)	2.342 ± 4.352 (2.332 ± 4.352)	2.103 ± 3.619 (2.099 ± 3.613)	9.150 ± 11.147 (9.037 ± 11.095)	8.594 ± 10.870 (8.586 ± 10.847)	6.975 ± 8.753 (6.984 ± 8.744)	1.619 ± 2.246 (1.602 ± 2.231)	5.407 ± 3.514 (5.477 ± 3.639)
All Sample Aggregate Av.^n, g^		16.28	99.958	1.715	2.463	1.340 (1.300)	1.303 (1.262)	1.950 (1.990)	1.716 (1.753)	7.427 (7.370)	6.912 (6.913)	5.520 (5.539)	1.392 (1.373)	3.967 (4.033)
Lung-to-Liver (WGS)^g^	M	1422.89	99.823	6.391	11.328	0.049 (0.045)	0.049 (0.045)	0.008 (0.008)	0.008 (0.008)	0.167 (0.167)	0.155 (0.155)	0.148 (0.149)	0.007 (0.006)	22.790 (22.952)
Liver (WGS)^g^	M	2601.55	99.892	4.780	5.992	0.190 (0.194)	0.188 (0.192)	0.016 (0.014)	0.005 (0.003)	0.144 (0.137)	0.121 (0.130)	0.103 (0.115)	0.017 (0.015)	5.974 (7.601)

^a^See Additional file 3: Table S1 and Additional file 1: Table S2 for data on individual cancer samples

^b^‘Sample’ refers in rows 1–38 (not counting row of headings) to 30 tumor-control pairs analyzed by AluScan as described in Methods; refers in row 39 to the lung-to-liver metastatic cancer analyzed by Ju et al. [8] using WGS; and refers in row 40 to the primary liver cancer analyzed by Ouyang et al. [9] using WGS

^c^Only nucleotide positions that were captured in both the tumor and the control samples for AluScan sequencing were analyzed and counted

^d^Mutation rates (R) were given in each instance by the ratio [100 % × (number of mutated residues) / (total number of residues analyzed)]. R_GOH-M_ refers to the % of MM residues, and R_GOH-m_ the % of mm residues, that underwent a GOH mutation. R_Mm_ refers to the % of Mm residues that underwent a mutation. R_LOH_ refers to the % of Mm residues that underwent an LOH mutation. The rates of LOHs leading to the production of MM residues and mm residues are given by R_Mm->MM_ and R_Mm->mm_ respectively

^e^The ratio between the MM residues and mm residues produced from Mm residues by LOH is expressed by MM/mm

^f^The average of the individual values for 5 leukemia samples ± SD

^g^All Av. values estimated without removal of CNV-associated LOHs and GOHs are shown without parentheses; all Av. values estimated after removal of CNV-associated LOHs and GOHs are shown inside parentheses

^h^Aggregate Av. for Total (Mb) represents 0.2 × total Mb analyzed in the 5 leukemia samples. Aggregate Av. values for the other columns are obtained directly from dividing by 5 the total figures for the 5 leukemia samples pooled together

^i, j^Sample Av. and Aggregate Av. values are defined as in f and h, but pertain to the 25 solid tumors

^k, l^Sample Av. and Aggregate Av. values are defined as in f and h, but pertain to the 5 glioma tumors

^m, n^Sample Av. and Aggregate Av. values are defined as in f and h, but pertain to all 30 cancer samples

### Inter-Alu PCR and next-generation sequencing

Next-generation sequencing technologies have transformed genetics through their ability to produce giga-bases of sequence information in a single run. However, next-generation sequencing of a subset of the genome captured by inter-Alu PCR with an amplicon range vastly enhanced by the use of both ‘head type’ and ‘tail type’ Alu consensus sequence-based PCR primers of opposing orientations could substantially reduce the amount of sample DNA required as well as data analysis [7]. Such sets of next-generation sequenced inter-Alu PCR amplicons, or AluScans, were employed in this study to provide an expedited scan of the mutations in exons, introns and non-coding regions. For this purpose, a 25-μl PCR reaction mixture contained 2 μl Bioline 10× NH_4_ buffer (160 mM ammonium sulfate, 670 mM Tris–HCl, pH 8.8, 0.1 % stabilizer; www.bioline.com), 3 mM MgCl_2_, 0.15 mM dNTP mix, 1 unit Taq polymerase, 0.1 μg DNA sample, and 0.075 μM each of the four following Alu-based PCR primers: AluY278T18 (5′-GAGCGAGACTCCGTCTCA-3′); AluY66H21 (5′-TGGTCTCGATCTCCTGACCTC-3′); R12A/267 (5′-AGCGAGACTCCG-3′) and L12A/8 (5′-TGAGCCACCGCG-3′). DNA denaturation was carried out at 95 °C for 5 min, followed by 30 cycles each of 30 s at 95 °C, 30 s at 50 °C, and 5 min at 72 °C, and finally another 7 min at 72 °C. Amplicons were purified with ethanol precipitation, sequenced on the Illumina-Solexa platform at Beijing Genomics Institute (Shenzhen, China) and mapped to the reference human genome hg19 [10].

### AluScan sequencing data mapping and variant analysis

Paired-end sequencing reads were aligned to the GRCh37.p2 (Feb 2009) reference human genome using BWA (Burrows-Wheeler Aligner, version 0.6.1) with default settings [11]. After BAM format-transfer and sorting by SAMtools (Sequence Alignment/Map, version 0.1.18) [12], the reads were further recalibrated and locally realigned using GATK (Genome Analysis Tool-Kit, version Lite-2.1-8-gbb7f038) [13] according to the standard framework [14].

The module ‘UnifiedGenotyper’ in GATK was employed to perform genotyping, and LOH and GOH callings for each sample were conducted with default settings. An LOH was defined as the conversion of a locus from heterozygosity in control to homozygosity in tumor, whereas a GOH was defined as the conversion of a locus from homozygosity in control to heterozygosity in tumor. Regions of read depths < 8 in either the tumor sample or its paired control would not be analyzed further. For homozygous reference loci, allele frequency must be 100 %. For homozygous non-reference loci, non-reference allele frequency must be 100 % with QD ≥ 20. For heterozygous loci, the non-reference allele must be ≥ 35 % and ≤ 65 % with Quality by Depth (QD) ≥ 4. Based on these conditions, with a ≥ 8 read depth, recognition of any site on the control sequence as a heterozygous site required a minimum of three reads bearing the non-reference allele. Since LOH would be called at this site on the tumor sequence only when the homozygous genotype was observed to be 100 %, an LOH event would be scored only if all these three non-reference allele reads in the control were no longer observed. Strand bias filter was employed to ensure SB values < −0.01 for both heterozygous loci and homozygous non-reference loci. See Additional file 2: Methods for more details on methods.

### CNV Analysis

A variety of algorithms have been designed for CNV calling from whole genome sequencing (WGS) or exome sequencing data. However, the special features of AluScan data rendered difficult the calling of CNV using the calling algorithms designed for WGS or exome sequencing. Accordingly, the AluScanCNV method developed by us to call CNVs from AluScan and other types of sequence data, based on Geary-Hinkley transformation of read-depth ratios between either paired test-control samples or between test samples and a reference template constructed from reference samples [15], was employed in the present study for the identification of CN-gains and CN-losses.

### Genic locations of mutations

The possible genic locations of the called LOH and GOH loci were identified through comparison with the Ensembl gene list from the UCSC database (http://genome.ucsc.edu/cgi-bin/hgTables), the TSGene database (Tumor Suppressor Gene: http://bioinfo.mc.vanderbilt.edu/TSGene/), and the NCG4.0 database (Network of Cancer Genes: http://ncg.kcl.ac.uk/).

### Variant analysis of whole genome sequencing data

Raw whole genome sequencing data for a lung-to-liver metastatic cancer from a lung adenocarcinoma [8], and a hepatitis B positive hepatocellular carcinoma [9] were aligned to the GRCh37.p2 (Feb 2009) reference human genome by using BWA and GATK to extract all the overlapping sites between blood cell control and tumor tissue in the case of the lung-to-liver metastatic cancer, and between normal liver tissue control and tumor tissue in the case of the primary liver cancer. Genotyping and variant callings were performed for each of these two DNA samples with the ‘UnifiedGenotyper’ module in GATK with default settings as described for the AluScan samples. Regions of read depths <15 were first filtered out. Further filtration was achieved using the following criteria: for the homozygous reference loci, allele frequency must be 100 % and Quality by Depth (QD) ≥ 1; for heterozygous loci, non-reference allele frequency must be ≥ 25 % and ≤ 75 % with QD ≥ 4; for homozygous non-reference loci, non-reference allele frequency must be 100 % with QD ≥20. Phred-scaled *p*-value using Fisher’s exact test to detect strand bias (FS) was employed to ensure FS value ≤ 12 for both heterozygous loci and homozygous non-reference loci. The percentile rate of LOH (or GOH) was estimated by dividing the number of LOHs (or GOHs) by the number of total Mm sites (or MM or mm sites) that had passed through the filtration steps described above, and multiplying by 100 %.

## Results

### High rate of LOH occurrence

The genome-wide single base-resolution LOHs detected in thirty primary and secondary cancers using the AluScan platform are summarized in Table 1 and Additional file 3: Table S1. In these tables, any allele at a residue in the genome that corresponded to the allele represented in the reference human genome hg19 was regarded as M, *viz*. the reference or major allele; on the other hand, any allele that differed from the allele on hg19 was regarded as m, *viz*. the minor allele. On this basis, the results in the tables showed that the heterozygous Mm residues in the genomes of gastric cancers, gliomas, leukemias, liver cancers and primary and secondary lung cancers all displayed exceptionally high mutation rates leading to an all-sample R_Mm_ equal to 9.15 % of all Mm residues analyzed, with a great majority of the mutations giving rise to LOH to yield an R_LOH_ of 8.59 %. In contrast, R_GOH-M_ for gain-of-heterozygosity was merely 1.54 × 10^−3^ % of all MM residues analyzed.

Accordingly, based on the all-sample averages, the R_LOH_/R_GOH-M_ ratio for the thirty cancers was 8.59 % / 1.54 × 10^−3^ % = 5.58 × 10^3^. Because the number of analyzed residues (*viz*. base pairs that were mapped in both the paired cancer and control samples) varied with the cancer-control pair, aggregate average R_LOH_ was also estimated directly as the ratio [all LOHs detected] / [all Mm residues analyzed] in the thirty cancer-control pairs, and aggregate average R_GOH-M_ as the ratio [all GOH mutations of MM residues detected] / [all MM residues analyzed]: thereby the aggregate R_LOH_/R_GOH-M_ ratio for the 30 cancers was 6.91 % / 1.30 × 10^−3^ % = 5.32 × 10^3^. Thus, by either route, R_LOH_ was more than 5000-fold greater than R_GOH-M_. Given the far greater number of MM residues than Mm residues in genomic sequences, but far smaller R_GOH-M_ than R_LOH_ values, the total number of GOH-M mutations in the 30 cancers were of the same order of magnitude as the total number of LOH mutations (6360 and 8315 respectively) (Additional file 4: Table S3.31). The vastly unequal rates of LOH and GOH occurrences also rendered unlikely that the massive numbers of LOHs arose from technical errors, which would have produced LOHs and GOHs randomly at comparable rates. In fact, based on the use of high density whole genome SNP arrays, occurrence of copy number-neutral LOHs was also found to be frequent in gastrointestinal stromal tumors where contamination of tumor samples with normal cells was generally low, leading to the suggestion that the frequency of copy number-neutral LOHs might tend to be underestimated in solid tumors on account of the low percentages of tumor cells in the samples [4]. In the present study, as indicated in Methods, all MM or mm genotypes arising from Mm genotypes must be 100 % for them to be called as LOHs in order to minimize or obviate the effects of varied percentages of tumor cells in a tumor sample.

### Chromosomal distribution of LOH

**Fig. 1:**
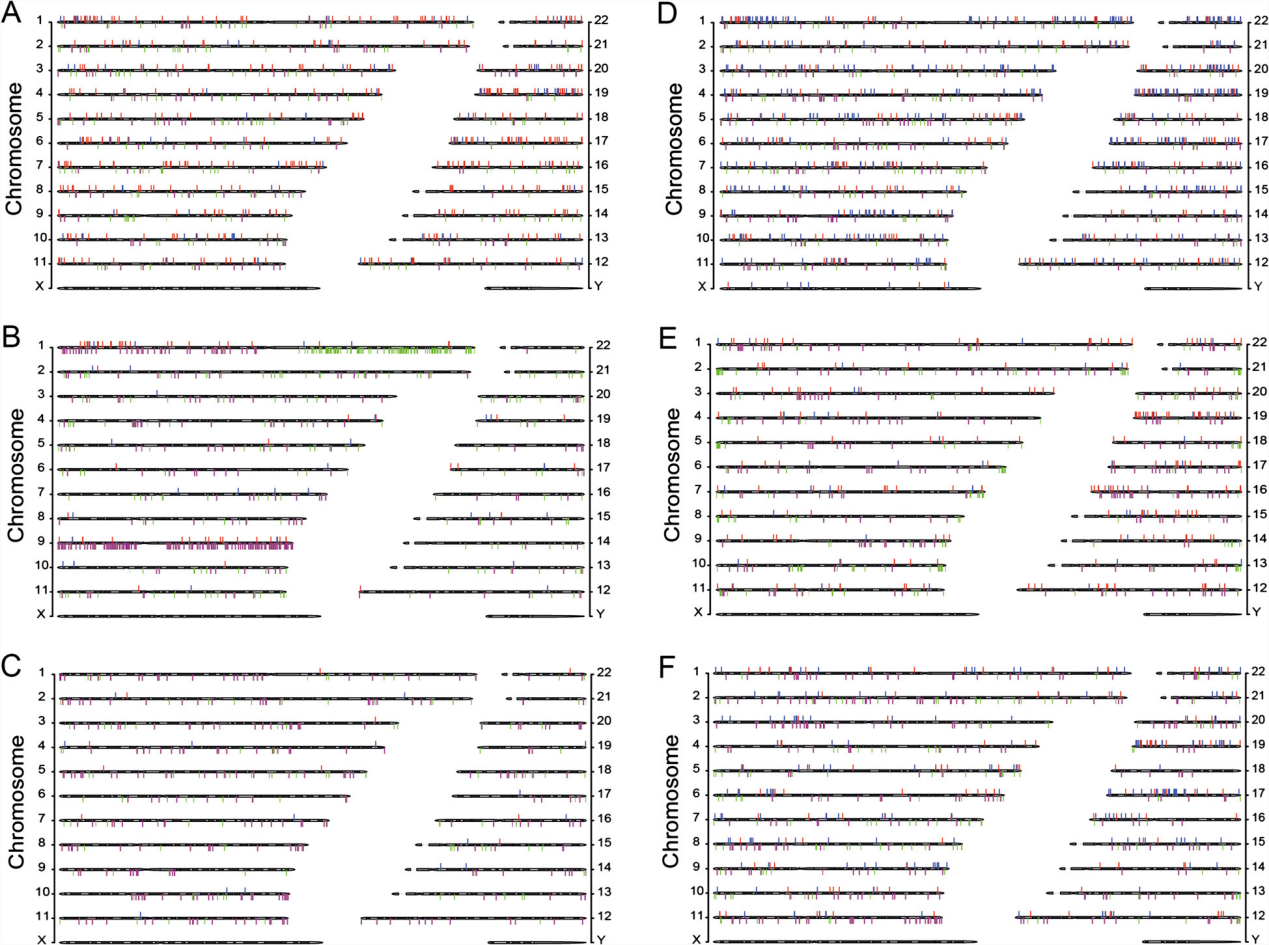
Chromosomal distributions of mutations in individual AluScan samples. LOHs (red bars) and GOHs (blue bars) detected from the AluScans of each tumor-control pair are plotted above the cytobands, and CN-gains (green bars) and CN-losses (purple bars) plotted below the cytobands, for six representative cancer samples. **a** Gastric 1, **b** Glioma 1, **c** Leukemia 1, **d** Liver 1, **e** Lung 1, and **f** Lung-Brain 1. Similar distributions for another twenty-four cancers are shown in Additional file 6: Figure S1. Complete listings of LOHs, GOHs and CNV sites in all thirty cancer samples, and comparison of the detected CNVs with those in the TCGA database [60] are given in Additional files 7: Table S4, Additional file 8: Table S5, Additional file 5: Table S6, respectively. Clinical information on each patient is indicated in the footnotes to Additional file 1: Table S2

**Fig. 2:**
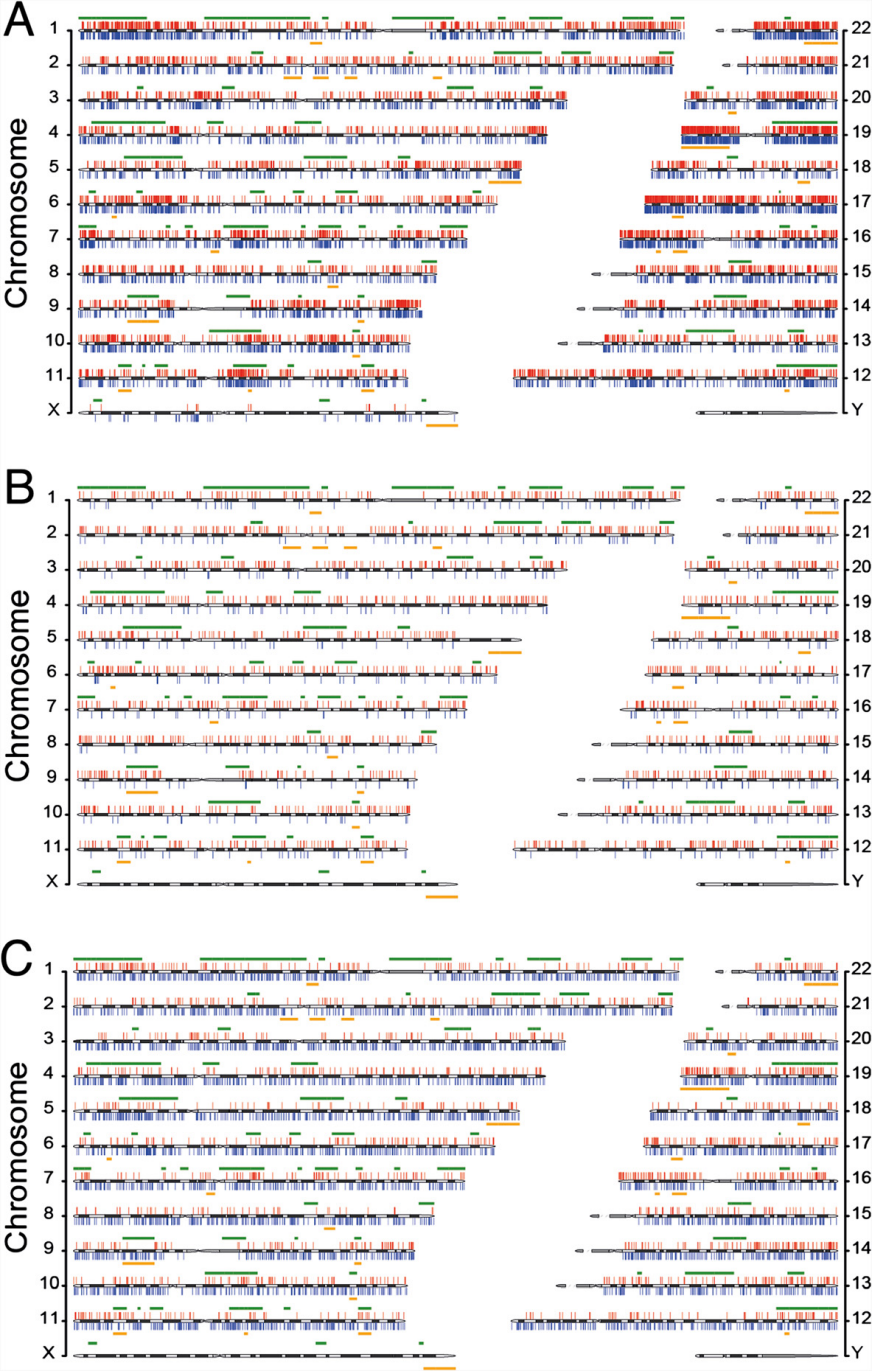
Chromosomal distributions of total mutations. **a** All LOHs and GOHs detected from AluScans of thirty cancer samples. **b** LOHs and GOHs detected from the whole genome sequences of a lung-to-liver metastatic cancer and its white blood cell control determined by Ju et al. [8]. **c** LOHs and GOHs detected from the whole genome sequences of a primary liver cancer and its normal liver tissue control determined by Ouyang et al. [9]. LOHs are shown as red vertical bars above cytobands, and GOHs as blue vertical bars below cytobands. The locations of common and rare fragile sites [19] are represented by horizontal green lines above, and horizontal orange lines below, the cytobands respectively. The chromosomal locations of all LOH and GOH sites are listed in Additional files 8: Table S5 and Additional files 5: Table S6

The LOHs detected in the thirty cancers analyzed by AluScan sequencing (Table 1) were mostly distributed over interstitial sites along the lengths of different chromosomes without extraordinary clustering at the ends of chromosomes (Figs. 1 and 2). This fundamentally interstitial character of cancer LOHs was readily discerned in the leukemias (e.g. Fig. 1c) where the majority of the chromosomes displayed sparse, isolated LOH occurrences. It was equally evident in chromosomal regions with a high density of LOHs, e.g. chr 19, chr 17, chr 1p and chr 22q in Fig. 2a, where dense LOHs were closely interspersed with dense GOHs, indicating that the dense LOHs could not be derived from long stretches of CN-losses which would be incompatible with the co-occurrence of dense GOHs within the same stretches.

Detection of CNV revealed some CNV-dense segments, e.g. CN-losses in chr 1p, chr 9p and chr 9q of Glioma 1 which coincided with frequently observed CN-losses in gliomas [16–18], and CN-gains in chr 1q of both Glioma 1 and Lung-Brain 4 as well as chr 19p of Glioma 4 (Fig. 1b, Additional file 5: Table S6; Additional file 6: Figure S1), but most of the LOHs observed in the cancers apart from the gliomas were copy-neutral ones unassociated with either CN-losses or CN-gains (Additional file 7: Table S4). Even for the glioma samples, the aggregate average R_GOH-M_ and R_LOH_ were only changed from 2.69 × 10^−3^ to 2.45 × 10^−3^ % and from 12.29 to 12.79 % respectively after subtraction of CNV-associated GOHs and LOHs (Table 1). Locations of common and rare fragile sites [19] overlapped some LOH-dense regions such as those in chr 1p, chr 11q, chr 12q, chr 19p, chr19q, and chr 22q, but not all LOH-dense regions (Fig. 2a).

In the lung-to-liver metastatic cancer and primary liver cancer analyzed by WGS sequencing, the LOHs detected were similarly found to be mainly interstitial along the lengths of chromosomes rather than concentrated near the ends of chromosomes (Fig. 2b, c). The GOHs were also thickly interspersed with the LOHs along various chromosomes, again ruling out extended segments of CN-losses as a major cause of the LOHs (Additional file 8: Table S5). While the AluScan results (Fig. 2a) showed some of the chromosomes such as chr 19, chr 17, and chr 22q to be particularly enriched with LOHs and GOHs, the LOHs and GOHs revealed by WGS were more uniformly distributed among different autosomal chromosomes, which is in agreement with the elevated density of Alu elements in chr 19 and chr 17 and therefore enhanced sequence capture from these chromosomes by AluScan (Additional file 4: Table S3.34). Earlier we also found single nucleotide variations to be elevated in the vicinity of Alu elements [20].

Notably, the R_LOH_/R_GOH-M_ ratio was ~640-3200 for the two sets of whole genome sequencing (WGS) data obtained by Ju et al. [8] and Ouyang et al. [9] (bottom two lines respectively, Table 1), compared to ~5300 for the all sample AluScan-based aggregate results. Therefore these two sequencing platforms were in agreement regarding the far greater percentile LOH mutations in cancer genomes compared to GOH mutations.

### Preferences for reference alleles and transitional changes in cancer LOHs

**Fig. 3:**
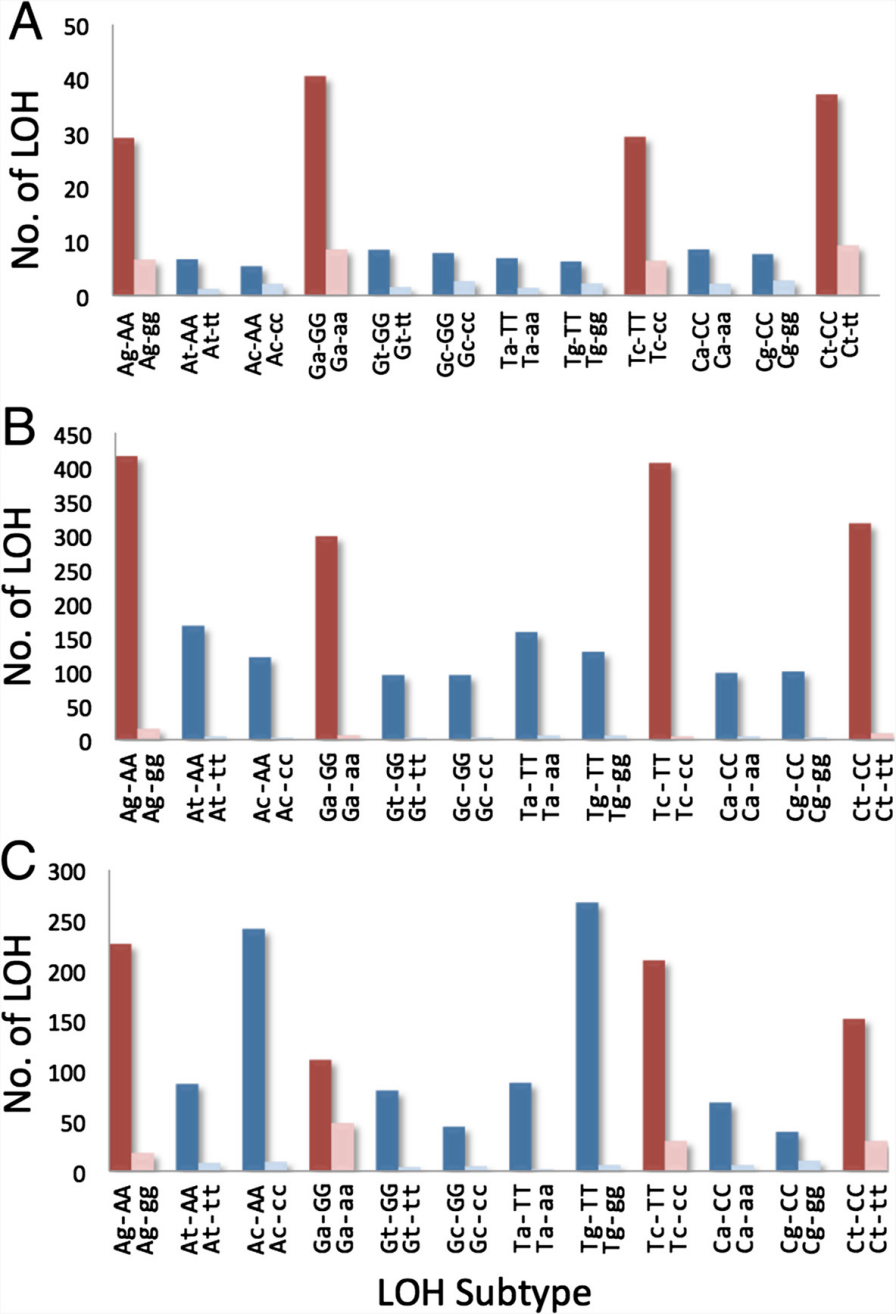
Preferences for reference alleles and transitional changes displayed by LOH mutations. The frequencies for the reference (i.e. major) allele versus the minor allele amongst the LOH products are shown by twin columns for transitional LOHs (red) and transversional LOHs (blue). The dark red and blue columns represent Mm-to-MM conversions (e.g. Ag-to-AA. indicated as Ag-AA), and the light red and blue columns represent Mm-to-mm conversions (e.g. Ag-to-gg indicated as Ag-gg). **a** Total preference profile of the thirty cancers in Table 1 analyzed by AluScan (obtained from the preference profiles of individual cancers given in Additional file 12: Figure S2). **b** Preference profile of the lung-to-liver metastatic cancer analyzed by WGS [8]. **c** Preference profile of the primary liver cancer analyzed by WGS [9]

In an LOH event, a heterozygous Mm residue with two different allelic bases on homologous chromosomes is mutated to a homozygous MM residue or a homozygous mm residue. In this regard, any residue in a human genome can be classified into the A-, G-, T- or C-family, depending on whether the reference allele at the same nucleotide position in the hg19 reference was an A, G, T or C. A preference for the reference (or M) allele represented by dark red or blue columns over the non-reference (or m) allele represented by light red or blue columns was observed for the LOHs arising from all 12 different types of Mm residues (Fig. 3a). Furthermore, transitional LOHs that produced the MM genotype (dark red columns), converting Ag to AA, Ga to GG, Tc to TT and Ct to CC, were far more prominent than transversional LOHs that produced the MM genotype (dark blue columns), converting At to AA, Ac to AA, Gt to GG, etc. Since the M-allele preference applied to LOHs arising from Mm residues belonging to all of the A-, G-, T- and C-families, it was distinct from GC-biased gene conversions [21].

M-allele preference was also displayed by the LOHs in the secondary lung-to-liver metastatic cancer [8] and the primary liver cancer [9] analyzed using WGS. Amongst the LOHs giving rise to MM genotypes in Fig. 3b, there was greater prominence of transitional LOHs than transversional LOHs as well. In Fig. 3c, the Ag-AA, Tc-TT and Ct-CC transitions remained prominent, but two of the transversions, *viz*. Ac-AA and Tg-TT, were likewise pronounced; whether this was related to the use of normal liver tissue as control in this instance remains to be determined. Thus the AluScan and WGS results were in accord with respect to M-allele preference, and both favored a prominence of transitional LOHs. A greater prominence of transitional relative to transversional LOHs is difficult to explain in terms of technical biases but consistent with the known higher transitional mutation rates relative to transversional mutation rates.

### Occurrence of LOH and GOH in cancer related genes

A large number of tumor suppressor genes and other cancer related genes in the AluScan sequence reads of the 30 cancer genomes, identified based on the TSGene and NCG4.0 databases, underwent LOH and GOH mutations (Additional file 9: Table S7). Table 2 shows the genes that displayed such mutations in multiple cancer samples, which would represent genes with a high likelihood to participate in the oncogenic pathways of the various cancers.

**Table 2:** Genes with LOH or GOH mutations in multiple cancer samples^a^

A. Genes in TSGene database with LOH occurrence in three or more cancer samples^b^			
**Chr** ^**c**^	**Gene**	**Description**	**Samples with LOH sites** ^**e**^
6	*AKAP12* ^d^	A kinase (PRKA) anchor protein 12	Gastric1, Gastric3, **Liver1**
6	*PARK2*	Parkin RBR E3 ubiquitin protein ligase	**Glioma3**, Glioma5, **Lung5**
7	*FBXL13*	F-box and leucine-rich repeat protein 13	Glioma3, **Liver1**, Lung5
7	*CUX1* ^d^	Cut-like homeobox 1	Glioma5, **Liver4**, Lung5, **Lung-Brain3**
8	*CSMD1* ^d^	CUB and Sushi multiple domains 1	Gastric1, Glioma2 (2), Glioma3 (2), Liver1, Liver3, **Liver5**, Lung1, Lung5, **Lung-Brain2** (**1** + 1)
8	*MFHAS1*	Malignant fibrous histiocytoma amplified sequence 1	Gastric1, Liver4, Liver5
9	*PPP2R4*	Protein phosphatase 2A activator, regulatory subunit 4	**Glioma1**, Lung3, Lung5
9	*PTPRD* ^d^	Protein tyrosine phosphatase, receptor type, D	**Glioma1**, Glioma3, Leukemia5, Liver1, Lung5 (3), Lung-Brain2
9	*ROR2*	Receptor tyrosine kinase-like orphan receptor 2	Gastric1 (3), Glioma3, Lung2
11	*NUP98* ^d^	Nucleoporin 98 kDa	**Gastric3**, Glioma3, Liver2, Lung-Brain1 (3)
12	*CHFR*	Checkpoint with forkhead and ring finger domains, E3 ubiquitin protein ligase	Glioma3, Liver2, Lung-Brain4
14	*EGLN3*	Egl-9 family hypoxia-inducible factor 3	**Lung5** (**1** + 1), Lung-Brain4, Lung-Brain5
16	*DNAJA3*	DnaJ (Hsp40) homolog, subfamily A, member 3	Liver4, Lung1, Lung5
16	*AXIN1* ^d^	Axin 1	Glioma2, Liver1, Lung5
19	*GLTSCR1* ^d^	Glioma tumor suppressor candidate region gene 1	Glioma5, Lung5, Lung-Brain4
22	*PRR5*	Proline rich 5 (renal)	Glioma3, **Liver3**, Lung5
**B. Additional genes present in NCG4.0 database with LOH occurrence in three or more cancer samples** ^**b**^			
**Chr** ^**c**^	**Gene**	**Description**	**Samples with LOH sites** ^**e**^
1	*SMG5*	SMG5 Nonsense Mediated MRNA Decay Factor	Liver2, **Lung1**, Lung5 (3), Lung-Brain2, Lung-Brain4
1	*KAZN*	Kazrin, Periplakin Interacting Protein	**Gastric3**, Liver1, Lung5, **Lung-Brain2**
2	*CTNNA2*	Catenin (Cadherin-Associated Protein), Alpha 2	Lung1, Lung3, **Lung5** (**2** + 2)
2	*DPP10*	Dipeptidyl-Peptidase 10 (Non-Functional)	Gastric1, **Glioma3**, Glioma5
3	*ERC2*	ELKS/RAB6-Interacting/CAST Family Member 2	Gastric3, Glioma3, Lung2
4	*ELOVL6*	ELOVL Fatty Acid Elongase 6	Glioma3, Glioma4, Lung-Brain2
4	*FSTL5*	Follistatin-Like 5	Glioma2, **Lung5**, Lung-Brain2
5	*AFF4*	AF4/FMR2 Family, Member 4	Gastric1, Glioma3, Lung5 (2)
5	*DMGDH*	Dimethylglycine Dehydrogenase	Gastric1, Liver5 (2), Lung2, Lung-Brain4
7	*HIP1*	Huntingtin Interacting Protein 1	**Gastric1**, Glioma5, Liver1, Liver4, Lung2, Lung5, **Lung-Brain2**
7	*CARD11*	Caspase Recruitment Domain Family, Member 11	**Glioma2**, Glioma5, Lung-Brain5
8	*RIMS2*	regulating synaptic membrane exocytosis 2	**Glioma3** (**1** + 1), **Lung5**, **Liver5**
9	*FNBP1*	Formin Binding Protein 1	Gastric5, Glioma5, Liver2 (2)
10	*SORCS1*	Sortilin-Related VPS10 Domain Containing Receptor 1	Gastric1, Glioma3, **Liver4**
10	*CTNNA3*	Catenin (Cadherin-Associated Protein), Alpha 3	Gastric1 (2), Glioma2, Lung-Brain1, Lung-Brain2
10	*PFKP*	Phosphofructokinase, Platelet	**Glioma3**, Lung5, Lung-Brain4
10	*TACC2*	Transforming, acidic coiled-coil containing protein 2	Gastric1, **Lung2**, Leukemia3, **Liver4**
10	*DLG5*	Discs, Large Homolog 5 (Drosophila)	Glioma5, Liver1, Lung5 (2)
10	*TET1*	Tet Methylcytosine Dioxygenase 1	Glioma3, Lung5 (4), **Lung-Brain2** (**1** + 1)
11	*NUMA1*	Nuclear Mitotic Apparatus Protein 1	**Glioma2**, Glioma4, Lung2
11	*PGAP2*	Post-GPI attachment to proteins 2	Lung4, Lung5, **Liver2**
13	*FREM2*	FRAS1 Related Extracellular Matrix Protein 2	Liver1, Lung2, Lung-Brain2
13	*ZMYM2*	Zinc Finger, MYM-Type 2	Gastric1, Liver1, Lung2
16	*SLC38A8*	Solute Carrier Family 38, Member 8	**Glioma3**, Glioma5, Lung-Brain2
16	*RBFOX1*	RNA Binding Protein, Fox-1 Homolog (C. Elegans) 1	Glioma3, **Liver4**, Lung5 (6)
17	*SEPT9*	Septin 9	Glioma1, Liver1, Lung5 (2)
17	*CDK12*	Cyclin-Dependent Kinase 12	Glioma5, Liver3, **Lung5** (**1** + 2)
17	*DNAH9*	Dynein, axonemal, heavy chain 9	**Glioma3**, Lung5 (2), **Lung-Brain1**, Lung-Brain4
17	*GAS7*	Growth Arrest-Specific 7	Gastric3 (2), Glioma2, Glioma3, Glioma4, **Liver2**, Lung-Brain4
17	*RPS6KB1*	Ribosomal Protein S6 Kinase, 70 kDa, Polypeptide 1	Gastric1, Liver2 (3), Liver4, Lung5
17	*WIPF2*	WAS/WASL Interacting Protein Family, Member 2	Liver2, Lung4, Lung-Brain2
18	*MBP*	Myelin Basic Protein	Gastric1, Glioma4, Lung5
18	*LAMA1*	Laminin, Alpha 1	Glioma3, **Liver4**, Lung5
18	*LDLRAD4*	Low Density Lipoprotein Receptor Class A Domain Containing 4	**Gastric4**, Glioma2, Glioma5, Lung5 (2), **Lung-Brain4**
18	*GREB1L*	Growth Regulation By Estrogen In Breast Cancer-Like	Liver4, Lung5, **Lung-Brain2**
19	*GLTSCR1*	Glioma Tumor Suppressor Candidate Region Gene 1	Glioma5, Lung-Brain4, Lung5
20	*ZSWIM3*	Zinc Finger, SWIM-Type Containing 3	Gastric1, Glioma3, Glioma5, Liver1
22	*TRIOBP*	TRIO And F-Actin Binding Protein	**Glioma5**, **Lung5** (**3**), Lung-Brain2
**C. Genes present in TSGene database with GOH occurrence in three or more cancer samples** ^**b**^			
**Chr** ^**c**^	**Gene**	**Description**	**Samples with GOH sites**
8	*CSMD1* ^f^	CUB and Sushi multiple domains 1	Gastric3 (2), Gastric4, Glioma3 (2), Lung5 (2)
9	*PTPRD* ^f^	Protein Tyrosine Phosphatase, Receptor Type, D	Glioma1, Liver1, Lung5, Lung-Brain5
9	*DAPK1*	Death-Associated Protein Kinase 1	Glioma4, Liver1, Lung1
9	*ROR2*	Receptor Tyrosine Kinase-Like Orphan Receptor	Gastric5, Glioma5, Lung5 (2)
11	*ST5*	Suppression of Tumorigenicity 5	Glioma3, Glioma4, Glioma5
12	*CHFR*	Checkpoint with forkhead and ring finger domains, E3 ubiquitin protein ligase	Gastric1, Glioma5, Lung5 (3), Lung-Brain3
16	*CDH1* ^f^	Cadherin 1, Type 1, E-Cadherin (Epithelial)	Gastric2 (3), Lung5 (3), Lung-Brain4
22	*CHEK2* ^f^	Checkpoint kinase 2	Glioma3, Lung2, Lung5
**D. Additional genes present in NCG4.0 database with GOH occurrence in three or more cancer samples** ^**b**^			
**Chr** ^**c**^	**Gene**	**Description**	**Samples with GOH sites**
1	*KAZN*	Kazrin, Periplakin Interacting Protein	Gastric4, Glioma4, Lung5 (4)
1	*FMN2*	Formin 2	Lung5, Liver1, Lung-Brain1
1	*NLRP3*	NLR Family, Pyrin Domain Containing 3	Glioma3 (3), Liver1 (2), Lung5
4	*FSTL5*	Follistatin-Like 5	Liver4, Lung2, Lung5 (2)
5	*PCDHGC5*	Protocadherin Gamma Subfamily C, 5	Liver2, Lung2, Lung5 (2)
7	*KMT2C*	Lysine (K)-Specific Methyltransferase 2C	Gastric2, Gastric5 (2), Leukemia4, Liver2, Liver4
9	*FNBP1*	Formin Binding Protein 1	Glioma5, Lung5, Lung-Brain1
9	*TRPM6*	Transient Receptor Potential Cation Channel, Subfamily M, Member 6	Glioma3, Liver4, Lung5
10	*CTNNA3*	Catenin (Cadherin-Associated Protein), Alpha 3	Glioma3, Liver1, Lung2, Lung5
10	*PFKP*	Phosphofructokinase, Platelet	Gastric1, Glioma1, Lung5
12	*SP1*	Sp1 Transcription Factor	Glioma3, Glioma4, Lung4 (2)
12	*KDM2B*	Lysine (K)-specific demethylase 2B	Glioma5, Lung5, Lung-Brain4
12	*ERC1*	ELKS/RAB6-Interacting/CAST Family Member 1	Gastric1, Lung2, Lung5, Liver5
13	*ATP11A*	ATPase, class VI, type 11A	Glioma3, Liver1, Liver2
16	*CNOT1*	CCR4-NOT Transcription Complex, Subunit 1	Glioma2, Glioma3, Lung5
16	*SNX29*	Sorting nexin 29	Gastric5, Lung5 (3), Liver1, Lung-Brain2
16	*RBFOX1*	RNA Binding Protein, Fox-1 Homolog (C. Elegans) 1	Gastric2, Glioma3 (4), Liver1 (2), Liver2, Lung4, Lung5 (3)
17	*SEPT9*	Septin 9	Liver1, Lung3, Lung5
17	*RPS6KB1*	Ribosomal Protein S6 Kinase, 70 kDa, Polypeptide 1	Gastric3, Glioma4, Lung-Brain3
17	*TRIM37*	Tripartite Motif Containing 37	Glioma1 (2), Glioma3, Glioma4
17	*ITGAE*	Integrin, Alpha E (Antigen CD103, Human Mucosal Lymphocyte Antigen 1; Alpha Polypeptide)	Glioma3, Glioma4 (2), Glioma5, Lung5 (2)
18	*GREB1L*	Growth Regulation By Estrogen In Breast Cancer-Like	Lung2, Lung4, Lung-Brain2 (2)
19	*PTPRS*	Protein tyrosine phosphatase, receptor type, S	Gastric1, Glioma5, Lung4
22	*TUBA8*	Tubulin, alpha 8	Gastric5, Glioma3, Lung5
**E. Additional genes present in Ensemble database with extensive LOH occurrences** ^**g**^			
	**Gene**	**Description**	**Samples with LOH sites** ^**e**^
2	*MTA3*	Metastasis associated 1 family, member 3 [Source:HGNC Symbol;Acc:23784]	**Gastric1,** Lung-Brain2, Lung3 (2), Liver2, Lung-Brain3
3	*EIF2B5*	Eukaryotic translation initiation factor 2B, subunit 5 epsilon, 82 kDa [Source:HGNC Symbol;Acc:3261]	**Glioma3**, Lung5 (8), **Liver5**, Leukemia1
4	*SMIM14*	Small integral membrane protein 14 [Source:HGNC Symbol;Acc:27,321]	Liver2, Lung-Brain5, Glioma3, Leukemia5, Lung1 (2)
6	*ATXN1*	Ataxin 1 [Source:HGNC Symbol;Acc:10548]	Glioma5, Lung4, Lung5, Liver1, **Lung-Brain5**
6	*RP11*-*146I2.1*	Not Applicable	Glioma3, Lung3, Lung5, Liver2, **Lung-Brain4**
7	*COL26A1*	Collagen, type XXVI, alpha 1 [Source:HGNC Symbol;Acc:18038]	Gastric1, Lung1, Lung2, Liver1, Lung-Brain1
7	*TYW1B*	tRNA-yW synthesizing protein 1 homolog B (S. cerevisiae) [Source:HGNC Symbol;Acc:33908]	Gastric1, Glioma3, **Glioma5**, **Lung1**, Lung5
8	*LOXL2*	Lysyl oxidase-like 2 [Source:HGNC Symbol;Acc:6666]	Liver2 (2), Glioma3, Glioma4, Liver1, Liver3
8	*RP11*-*124B13.1*	Not Applicable	**Gastric1**, Glioma1, Glioma3, **Lung2**, Liver2
9	*DMRT1*	Doublesex and mab-3 related transcription factor 1 [Source:HGNC Symbol;Acc:2934]	Glioma5, Gastric3, Lung5, Glioma1, **Gastric1**, Lung-Brain2
9	*ODF2*	Outer dense fiber of sperm tails 2 [Source:HGNC Symbol;Acc:8114]	Lung-Brain2, **Lung5** (**2**), Glioma5, Lung4, Lung-Brain4, Liver4
10	*CAMK1D*	Calcium/calmodulin-dependent protein kinase ID [Source:HGNC Symbol;Acc:19341]	Lung5 (4), Liver5, Glioma5 (2), Leukemia4, Lung-Brain4, **Liver1** (**1** + 1), Glioma3
10	*FRMD4A*	FERM domain containing 4A [Source:HGNC Symbol;Acc:25491]	**Lung5** (**2** + 8), Glioma5, Glioma3, **Gastric1**, Liver5
11	*TMEM135*	Transmembrane protein 135 [Source:HGNC Symbol;Acc:26167]	Gastric1, Glioma3, Glioma4, Glioma5, Lung3
14	*KLC1*	Kinesin light chain 1 [Source:HGNC Symbol;Acc:6387]	Liver5, Lung-Brain2, Gastric1, Glioma3, Liver2, Lung5
16	*ABCC1*	ATP-binding cassette, sub-family C (CFTR/MRP), member 1 [Source:HGNC Symbol;Acc:51]	**Lung5** (**2** + 2), **Lung1**, Liver2, **Lung-Brain2**, Lung-Brain1
16	*C16orf45*	Chromosome 16 open reading frame 45 [Source:HGNC Symbol;Acc:19213]	**Gastric1**, **Glioma3**, Lung2, Lung5, Lung-Brain5
17	*NMT1*	N-myristoyltransferase 1 [Source:HGNC Symbol;Acc:7857]	Glioma5, Liver4, Lung5 (2), Gastric1 (2), Glioma3,
17	*PITPNC1*	Phosphatidylinositol transfer protein, cytoplasmic 1 [Source:HGNC Symbol;Acc:21045]	Gastric1, **Gastric4**, Glioma3, Glioma5, Lung2
17	*RAP1GAP2*	RAP1 GTPase activating protein 2 [Source:HGNC Symbol;Acc:29176]	Liver2 (2), **Glioma5** (**2)**, **Lung-Brain2** (**2** + 1), Gastric5, Gastric1, Lung4, Lung5
17	*RBFOX3*	RNA binding protein, fox-1 homolog (C. elegans) 3 [Source:HGNC Symbol;Acc:27097]	Glioma3, Glioma5, Liver3, **Lung-Brain4** (**1** + 3), Lung-Brain5
18	*DLGAP1*	Discs, large (Drosophila) homolog-associated protein 1 [Source:HGNC Symbol;Acc:2905]	Lung-Brain2, **Lung5** (**2** + 1**)**, Gastric4, **Gastric1**, Lung-Brain2, Glioma3
19	*INSR*	Insulin receptor [Source:HGNC Symbol;Acc:6091]	**Liver3**, Liver4, Lung2, **Lung5** (**1** + 1), Gastric1 (2)
19	*SIPA1L3*	Signal-induced proliferation-associated 1 like 3 [Source:HGNC Symbol;Acc:23801]	Glioma3, Lung-Brain2, Liver2, Lung5, Glioma3, Lung5
19	*TDRD12*	Tudor domain containing 12 [Source:HGNC Symbol;Acc:25044]	Lung-Brain1, Glioma5, Lung1, Glioma5, **Glioma2**, Liver2
19	*GNG7*	Guanine nucleotide binding protein (G protein), gamma 7 [Source:HGNC Symbol;Acc:4410]	**Lung5** (**1** + 7), **Lung-Brain4** (**3**)
22	*SYN3*	Synapsin III [Source:HGNC Symbol;Acc:11496]	Lung3, Lung5, Lung-Brain4, **Glioma3** (**1** + 2), Glioma5
**F. Additional genes present in Ensemble database with extensive GOH occurrences** ^**g**^			
**Chr** ^**c**^	**Gene**	**Description**	**Samples with GOH sites** ^**e**^
1	*RPRD2*	Regulation of nuclear pre-mRNA domain containing 2 [Source:HGNC Symbol;Acc:29039]	Gastric2, Glioma3, Glioma4, Glioma5 (2), Liver1, Lung4, Lung5
1	*CLSTN1*	Calsyntenin 1 [Source:HGNC Symbol;Acc:17447]	Gastric5, Lung2, Lung5, Lung-Brain2, Lung-Brain4
1	*KIF26B*	Kinesin family member 26B [Source:HGNC Symbol;Acc:25484]	Liver3, Glioma3, Glioma5 (2), Lung5 (3), Gastric1
1	*GON4L*	Gon-4-like (C. elegans) [Source:HGNC Symbol;Acc:25973]	Lung2 (2), Lung5 (2), Glioma4 (2), Lung-Brain1, Gastric4
1	*NMNAT2*	Calsyntenin 1 [Source:HGNC Symbol;Acc:17447]	Glioma5, Lung1, Lung5, Liver1, Lung-Brain1
2	*FAM178B*	Family with sequence similarity 178, member B [Source:HGNC Symbol;Acc:28036]	Gastric5, Glioma3, Liver3, Liver5, Lung3
2	*MTA3*	Metastasis associated 1 family, member 3 [Source:HGNC Symbol;Acc:23784]	Glioma1, Glioma2, Glioma4, Liver1, Lung-Brain3
3	*EIF2B5*	Eukaryotic translation initiation factor 2B, subunit 5 epsilon, 82 kDa [Source:HGNC Symbol;Acc:3261]	Gastric3, Glioma5 (3), Lung5, Lung-Brain2, Lung-Brain4
4	*AFAP1*	Actin filament associated protein 1 [Source:HGNC Symbol;Acc:24017]	Glioma3 (2), Liver1, Lung5 (6), Leukemia4, Liver4, Glioma5
5	*PDZD2*	PDZ domain containing 2 [Source:HGNC Symbol;Acc:18486]	Lung2, Lung5 (4), Glioma1, Gastric2, Glioma3 (2)
5	*PCDHGA1*	Protocadherin gamma subfamily A, 1 [Source:HGNC Symbol;Acc:8696]	Glioma3, Glioma5, Liver2, Lung2, Lung5 (2)
7	*CALN1*	Calneuron 1 [Source:HGNC Symbol;Acc:13248]	Glioma3, Glioma3, Lung2 (5), Lung4, Lung5 (2)
7	*KMT2C*	Protocadherin gamma subfamily A, 1 [Source:HGNC Symbol;Acc:8696]	Gastric2, Gastric5 (2), Liver2, Liver4, Leukemia4
7	*TYW1*	tRNA-yW synthesizing protein 1 homolog (S. cerevisiae) [Source:HGNC Symbol;Acc:25598]	Glioma3, Glioma5 (3), Liver1, Lung-Brain1 (5)
8	*PSD3*	Pleckstrin and Sec7 domain containing 3 [Source:HGNC Symbol;Acc:19093]	Lung3, Lung5 (9)
10	*CAMK1D*	Calcium/calmodulin-dependent protein kinase ID [Source:HGNC Symbol;Acc:19341]	Glioma3, Liver1, Lung3, Lung4 (5), Lung5 (5), Glioma4
11	*SHANK2*	SH3 and multiple ankyrin repeat domains 2 [Source:HGNC Symbol;Acc:14295]	Glioma3, Lung3, Lung5, Liver1, Liver5
12	*MPHOSPH9*	M-phase phosphoprotein 9 [Source:HGNC Symbol;Acc:7215]	Leukemia3, Lung-Brain5, Lung2, Lung4 (2), Lung5, Glioma4
17	*STX8*	Syntaxin 8 [Source:HGNC Symbol;Acc:11443]	Glioma3, Glioma4 (2), Lung2, Lung4 (2), Leukemia2
17	*USP43*	SH3 and multiple ankyrin repeat domains 2 [Source:HGNC Symbol;Acc:14295]	Liver1, Lung2, Lung5, Glioma5, Lung-Brain4
19	*INSR*	Insulin receptor [Source:HGNC Symbol;Acc:6091]	Liver4, Glioma3 (2), Glioma4 (2), Lung-Brain3, Lung5 (3)
19	*CTC*-*490E21.12*	Not Applicable	Gastric2, Gastric3, Glioma4, Glioma5, Lung1
19	*CTC*-*525D6.1*	Not Applicable	Gastric2 (4), Lung5 (8)
19	*SAE1*	SUMO1 activating enzyme subunit 1 [Source:HGNC Symbol;Acc:30660]	Glioma3, Lung2, Lung4, Lung5, Liver1
20	*ATP9A*	ATPase, class II, type 9A [Source:HGNC Symbol;Acc:13540]	Liver1 (3), Glioma4, Gastric3, Lung4, Lung5 (2)
22	*LARGE*	Like-glycosyltransferase [Source:HGNC Symbol;Acc:6511]	Liver1 (2), Lung1, Lung2, Leukemia5 (2), Glioma5 (3), Gastric5 (2)
22	*SGSM1*	Lysine (K)-specific methyltransferase 2C [Source:HGNC Symbol;Acc:13726]	Gastric2, Glioma3, Leukemia5, Lung-Brain2 (2), Lung-Brain3

^a^See Additional file 9: Table S7A-F for complete lists of genes with LOH or GOH occurrence

^b^TSGene: Tumor Suppressor Gene Database, containing 860 genes; NCG4.0: Network of Cancer Genes, containing 2000 genes

^c^ Chromosome on which the indicated gene is located

^d^LOH-containing genes present in both TSGene and NCG4.0 databases are only listed in Table 2A but not Table 2B

^e^The bold-fonted LOH occurrences represent Mm-to-mm conversions, and the non-bold-fonted LOH occurrences represent Mm-to-MM conversions. Where a sample contained more than one LOH, the number of LOHs is indicated inside parenthesis, either in bold font for LOHs yielding mm genotypes, or in non-bold font for LOHs yielding MM genotypes

^f^GOH-containing genes present in both TSGene and NCG4.0 databases are only listed in Table 2C but not Table 2D

^g^The list includes LOH- or GOH-bearing genes in Ensemble database (GRCh37.p13), which contains 57,736 genes, that are not in TSGene or NCG4.0

**Fig. 4:**
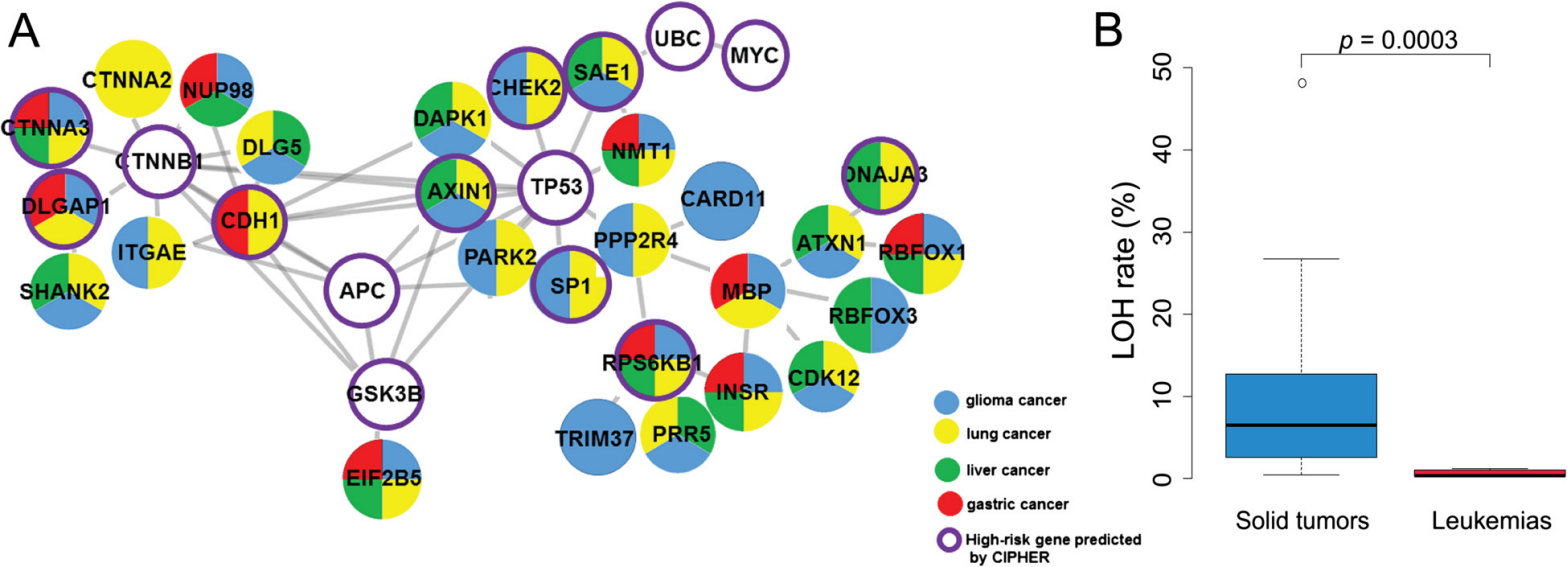
Mutations in solid tumors. **a** CIPHER analysis. Genes from Table 2 with multiple LOH or GOH mutations in gliomas, lung cancers, liver cancers and gastric cancers are marked in blue, yellow, green and red respectively, or multi-colored where such mutations were detected in more than one of these types of cancers. Genes identified by CIPHER to be high-risk for this group of cancers are enclosed by purple circle. **b** Comparison with leukemias in R_LOH_ rates. The sample average R_LOH_ rates of the twenty-five solid tumors and five leukemias are given in Table 1

To facilitate delineation of the genetic basis of human diseases, the bioinformatics tool CIPHER [22] has been developed to predict and prioritize disease genes based on the concordance between human protein network and disease phenotype network. Figure 4a shows an interaction network module associated with the solid tumor group, where the high-risk genes identified for this group by CIPHER based on database STRING [23] (Additional file 10: Table S8) interact with high likelihood genes from Table 2 that displayed multiple LOH and/or GOH mutations in the four solid tumor types (shown as color-coded sectors). Notably the genes *CTNNA3*, *DLGAP1*, *CDH1*, *CHEK2*, *SAE1*, *SP1*, *RPS6KB1*, *AXIN1* and *DNAJA3* were included in the high-likelihood genes in Table 2 as well as the CIPHER-identified high-risk genes. The network in Fig. 4a illustrates the utility of the identified multi-LOH/GOH genes in combination with CIPHER for analyzing potential protein-protein interactions in oncogenic networks.

Besides the multiple LOH- or GOH-bearing cancer-related genes detected, a host of other genes from the Ensembl database that were unlisted in either TSGene or NCG4.0 also underwent LOH and GOH mutations (Additional file 9: Tables S7E-F), and those unlisted genes that displayed highest occurrences of LOH or GOH, or in the largest numbers of cancer samples, are shown in Tables 2E-F, e.g. *FRMD4A* with fourteen LOHs, *CAMK1D* with 12 LOHs and 14 GOHs, *AFAP1* with twelve GOHs, and *LARGE* with eleven GOHs. Based on the multiplicity of LOH and/or GOH mutations displayed by these genes in the various cancer samples, they would represent potential cancer-related genes that merit further investigation: eight of these genes, *viz. DLGAP1*, *SHANK2*, *EIF2B5*, *SAE1*, *INSR*, *ATXN1* and *RBFOX1* are included in Fig. 4a along with 20 tumor-suppressor and cancer related genes from Table 2A-D. These findings underline the usefulness of comprehensive LOH and GOH tracking in multiple cancers for uncovering potential cancer-related genes.

## Discussion

On account of the complexity of cancer cells, genomic studies provide an excellent approach to find surprises [24]. In the present study, a characterization of the landscape of cancer LOHs revealed the surprisingly massive rates of LOH formation in various cancers, far exceeding the rates of GOH-M formation, and these cancer LOHs displayed a number of special properties.

### Features of cancer copy-neutral LOHs

#### Unequal incidences in solid tumors and leukemias

In Table 1, the average leukemia R_LOH_ of 0.61 % Mm residues was significantly lower than the average solid tumor R_LOH_ of 10.2 % Mm residues with *p* = 0.0003 (Fig. 4b), in accord with previous reports of lower mutation rates in leukemias compared to solid tumors [25, 26]. Furthermore, insofar that the leukemia and solid tumor samples were analyzed using the same procedures, the leukemia R_LOH_ of 0.61 % Mm residues suggests that the maximum technical error in the estimation of both leukemia and solid tumor R_LOH_ incurred by false-positive calling of LOHs would not exceed 0.61 % Mm residues, amounting to only 0.61/10.2, or 6 % error, for the solid tumors. At the other extreme, the exact causes for the extraordinary >20 % R_LOH_ rates of Gastric 1, Glioma 3, Glioma 5, Liver 1, and Lung 5 were undetermined; these cancers could be entering a terminal state of cellular disarray, and contribution from treatment modality-induced chromosomal instability also could not be ruled out.

#### Interstitial distribution

Among different chromosomes, dense LOHs were present over large portions of chr 19, chr 17, chr 16p, chr 22q, and parts of chr 1p, chr 6p, chr 9q and chr 11q, but were relatively sparse in chr 4, chr 8, chr 13, chr 18 and chr 21 (Fig. 2a). Some but not all of the dense LOH regions overlapped with the locations of known common or rare fragile sites. Association of LOHs with CNVs was evident in some instances, notably in Glioma 1, but such CNV-associated LOHs represented only a minor fraction of the LOHs observed (Fig. 1, Additional file 7: Table S4).

**Fig. 5:**
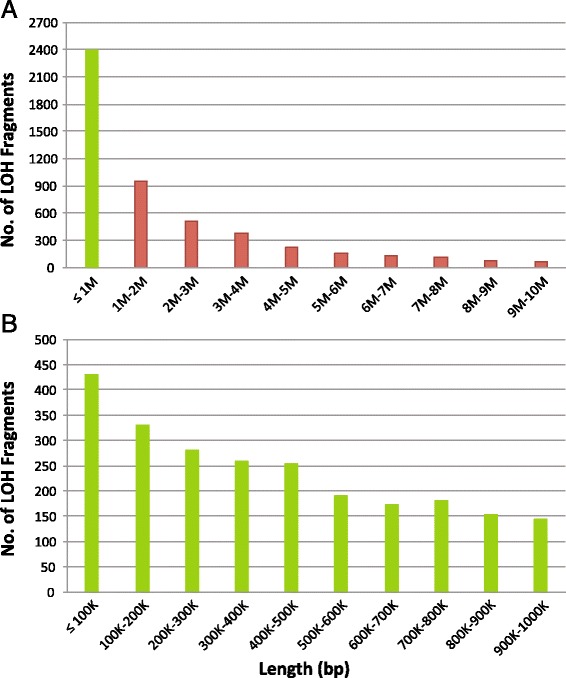
LOH fragment lengths. **a** Length distribution of total LOHs. **b** Expanded distribution of ≤ 1 Mb fraction of LOHs (green column in Part A). Distributions are based on total LOHs observed in the thirty tumors in Table 1 analyzed using AluScan. Individual fragment lengths are given in Additional file 11: Table S9

The mainly interstitial character of the cancer LOHs was evident from the well-spaced LOH occurrences in the sparse LOH regions of Fig. 2a and most regions in Fig. 2b and c, as well as the distribution of LOH fragment lengths showing a substantial fraction of fragments that were ≤ 1 Mb in size (Fig. 5; Additional file 11: Table S9). It was also evident in the LOH-dense regions in Fig. 2a, where the crowded interspersion of LOHs and GOHs was incompatible with the dense LOHs being the result of any extended stretches of CN-losses. The interstitial nature of the major fraction of copy-neutral LOHs observed supports gene conversion being an important mechanism in the production of cancer LOHs.

#### Reduced LOHs on X-chromosomes

**Fig. 6:**
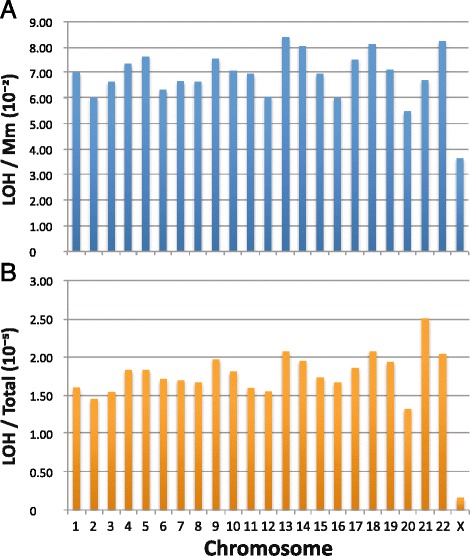
Relative abundance of LOH on different chromosomes. Estimates were based on the number of LOHs detected on each of the twenty-two autosomal chromosomes in the thirty cancer samples analyzed using AluScan in Table 1, and on the X chromosome in only the nine female cancer patients. **a** LOH per Mm site analyzed on each chromosome. **b** LOH per bp analyzed on each chromosome

R_LOH,_ the rate of LOH occurrence expressed as the percentile conversion of Mm residues analyzed, varied between different chromosomes (Fig. 6a). Among the autosomal chromosomes, this rate ranged from 5.5 % in chr 20 to 8.4 % in chr 13 (Additional file 4: Table S3.31). However, it was only 3.6 % in the X chromosomes in the female samples, which suggests that the inactive Barr-body configuration of one of the X-chromosomes [27] might constrain its participation in interhomolog recombination and LOH production relative to other chromosomes.

Interestingly, the four chromosomes exhibiting the highest levels of LOH/Mm, *viz*. chr 13, chr 22, chr 18 and chr 14, are known for their propensity to chromosomal instability as indicated by the frequency of trisomy [28, 29]. Also, the ~10^−5^ frequency of LOH occurrence on the various chromosomes expressed on a per bp basis (Fig. 6b) was similar to the 10^−6^ to 5 × 10^−5^ per bp frequencies displayed by TCGA ovarian cystadenoma and glioblastoma genomes [6].

#### Preferences for reference alleles and transitional changes

The partition of Mm genotypes between the MM and mm outcomes of LOH was characterized by a preference for MM over mm genotypes, with transitional LOHs being more prominent than transversional ones in the process (Additional file 12: Figure S2). Plausibly the M-alleles in the human genome were selected in the course of primate and human evolution to result in the adoption of MM genotypes at >99.9 % of the residues in the human genome. This bias in favor of the MM over Mm and mm genotypes suggests that, although the presence of m-alleles in the genome confers beneficial sequence diversity and gene-dosage modulation, functional advantages generally accrue to the M-alleles over m-alleles at most of the base positions in the genome. Accordingly, although key LOHs introducing mm-genotypes into tumor suppressor genes could facilitate oncogenesis [1], excessive accumulation of mm-genotypes might be detrimental to the cancer cells themselves, leading to selection against them and M-allele preference.

The taller column heights of transitional over transversional LOHs giving rise to MM-genotypes in Fig. 3a, b, and to a lesser extent in Fig. 3c, was in accord with the greater frequencies of transitional compared to transversional mutations in organisms.

### Mode of double strand break repair in cancers

Since random point mutations would yield similar rates of mutation of Mm residues to yield LOHs and MM residues to yield GOHs, the vastly higher R_LOH_ than R_GOH-M_ values in Table 1 rule out point mutations as an important mechanism for the generation of cancer LOHs. This together with the limited role of CNV focuses attention on repair of double strand breaks (DSB) as a major source of cancer LOHs. In eukaryotic cells, DSBs are repaired by a spectrum of mechanisms through non-homologous end-joining, and homologous recombinations (HR) that include crossover pathways, break-induced replication, and synthesis-dependent strand-annealing employing a repair template supplied by sister chromatid in inter-sister chromatid recombination (ISR), a homologous chromosome in interhomolog recombination (IHR), or some ectopic sequence to bring about gene conversion [30].

**Fig. 7:**
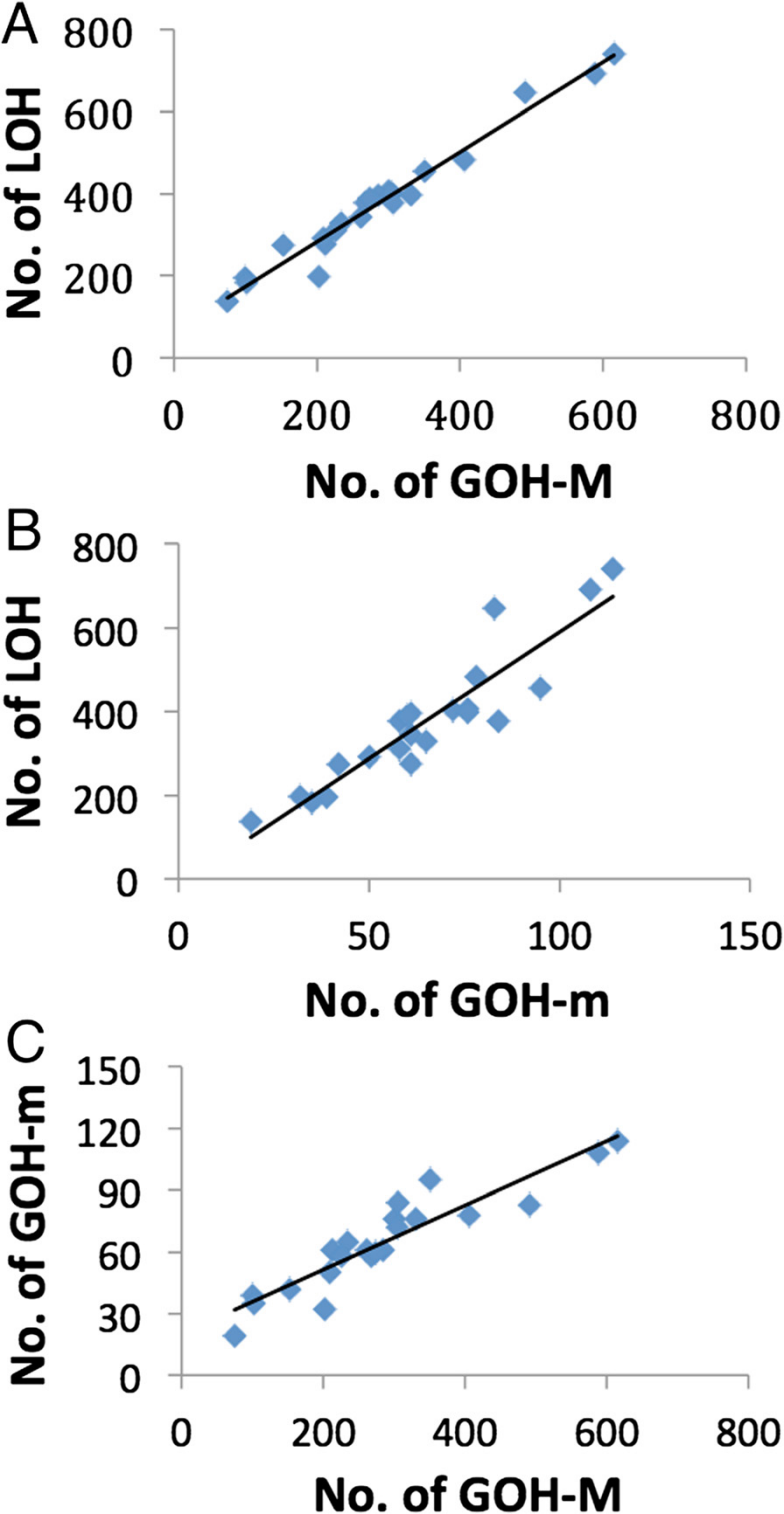
Linear correlations between three different types of mutations in cancer genomes. **a** Correlation between LOH and GOH-M, correlation coefficient =0.984. **b** Correlation between LOH and GOH-m, correlation coefficient = 0.920. **c** Correlation between GOH-m and GOH-M, correlation coefficient = 0.923. Each point represents the total number of each type of mutations detected on one of twenty-two autosomal chromosomes in all thirty cancer samples

Break-induced replication would be inconsistent with the largely interstitial character of the LOHs, and deletions of base residues at Mm sites due to non-homologous end-joining would not be called as SNVs (either LOHs or GOHs) in variant analysis (see Methods). In contrast, the HR process is known to produce relatively short patches of new DNA [31], in accord with the interstitial character of the cancer LOHs. Because ISR enhances both R_MM_ and R_LOH_ comparably whereas IHR enhances R_LOH_ far in excess of R_MM_, the finding of R_LOH_ > > R_GOH-M_ with the different types of cancers strongly favored IHR over ISR as the underlying mechanism for the massive cancer LOHs. This conclusion was supported by the linear correlations between the levels of LOH, GOH-M and GOH-m, where GOH-M denotes GOHs arising from homozygous MM residues, and GOH-m denotes GOHs arising from homozygous mm residues (Fig. 7). That the occurrence of GOH mutations in MM and mm tagged along in correlation with the occurrence of LOH mutations was to be expected with IHR, which would cause not only the Mm residues involved to undergo LOH mutation due to the use of the allelic template for DSB repair, but also significant mutations of the Mm, MM and mm residues involved owing to the highly error-prone nature of the DNA polymerase employed for invading strand elongation in the course of IHR, increasing the mutation rate by up to 1400-fold [32]. That R_GOH-m_ was also much greater than R_GOH-M_ would be consistent with the presence of extensive selection against mm-genotypes during cancer cell evolution, as suggested by the preference for M-alleles amongst the cancer LOHs.

The choice between ISR and IHR for DSB repair depends on a wide array of regulatory factors [33]. IHR prevails in meiotic cells. In mitotic cells, ISR prevails over IHR on account of the close positioning of sister chromatids secured by cohesin [34–36], but IHR can be increased upon induction of a chromosomal DSB by two to three orders of magnitude up to 1 % of the target sites to yield short tract nonreciprocal gene conversions [37]. Cellular systems are also known to undergo enhanced LOH production under special circumstances. In a Bloom mouse model, *Blm*-deficient ES cells exhibited elevated mitotic recombination rates with an 18-fold increase in somatic LOH [38]. In aging diploid mother yeast cells, recombination rates could increase to 200-fold the rate in young cells to result in an age-induced switch to a hyper-recombination state [39]. Thus the degenerative states in ageing yeast cells and the various types of tumors analyzed in Table 1 shared the common attribute of hyper-recombination. Interestingly, copy-neutral LOHs in ovarian cancer were found to be more frequent in older patients, suggesting that the effects of cancer and ageing could be additive in this regard [40].

The rate of DSB occurrence in cells has been estimated at about 50 DSBs per cell per cell cycle [41] or ten per day [42], and it is increased by both exogenous agents such as chemicals, ultraviolet and ionizing radiation, and endogenous events such as arrested replication forks, nucleases and reactive oxygen species from cellular metabolism [30, 43]. In the event that both leading and lagging strands of DNA are synthesized by discontinuous synthesis in human cells as has been suggested for *Escherichia coli* [44, 45], DNA synthesis itself can be a significant source of DSBs [33]. Evidence for oncogene-induced DSBs has been provided by using the presence of p53 binding protein 1 (53BP1) nuclear foci as indicator of DSBs, whereby 10–20 foci per cell could be detected in cancer cell lines but not in proliferating normal cells [46].

### Defective DNA-damage response

To cope with the continuous threat of DSBs, eukaryotic cells possess the capacity to mount a DNA-damage response (DDR) that arrests cell-cycle progression at the G_1_-S, intra-S and G_2_-M checkpoints to increase the time available for DNA repair; if the DNA damage cannot be removed, chronic DDR triggers cell death by apoptosis or cellular senescense [47–49]. Analysis of the relationship between DDR and oncogenesis has brought important insight into how oncogene activation-induced DNA hyper-replication could lead to S-phase DNA damage, onset of DDR and abrogation of cell cycle checkpoints, leading to a circumvention of the apoptosis and senescence pathways normally elicited by DDR, and hence oncogenesis [46, 50–52]. Notably, this chain of events, by diminishing or nullifying the action of the cell cycle checkpoints, not only would increase the influx of DSB-bearing DNA into the S-phase during the pre-oncogenesis phase to induce oncogenesis, but also may be expected to continue in the post-oncogenesis phase.

While usage of IHR for DSB repair is suppressed in favor of ISR in mitotic cells, the situation is radically altered upon oncogenesis and relaxation of checkpoints to enable the entry of DSB-bearing DNAs into S-phase. Under these circumstances, when a DSB-bearing chromatid replicates to yield a sister chromatid, the two sister chromatids will be unable to provide a useful repair template to one another. Instead, either a homologous chromosome or a homologous ectopic sequence will have to supply the requisite repair template [53]. Given the reliable presence of the homologous chromosome as template compared to the haphazard availability of a homologous ectopic sequence, DSB repair by IHR will prevail giving rise to hyper-accumulation of LOH mutations in the cancer cells. Although DSB repair by HR in general can be a source of mutations arising from error-prone polymerases or replication forks [32, 54, 55], in the presence of intact cell cycle checkpoints ISR will be strongly preferred over IHR for DSB repair during S-G_2_, such that the error-prone polymerases would bring about comparable R_GOH-M_ and R_LOH_ rates instead of the vastly higher R_LOH_ than R_GOH-M_ rates found in cancer samples. Therefore only impaired cell cycle checkpoints arising from a defective DDR can lead to the hugely greater R_LOH_ than R_GOH-M_ displayed by different types of cancer cells.

## Conclusions

The question has been posed regarding how might the requisite genetic changes, estimated to be about six mutations, be acquired to initiate oncogenesis, and whether mutator phenotypes participate in the process [56]. This question is important not only to oncogenesis, but also to the post-oncogenesis phase with respect to the mutations needed to implement the manifold hallmarks of the neoplastic state, i.e. sustaining proliferative signaling, evading growth suppressors, resisting cell death, enabling replicative immortality, inducing angiogenesis, and activating invasion and metastasis [57], and complex metabolic reprogramming to support rapid growth even under conditions of fluctuating oxygen tension through enhanced glucose uptake, aerobic glycolysis, decreased conversion of pyruvate to acetyl-CoA etc. [58]. Thus solid tumors compared to leukemias are confronted with irregular vascularization and more fluctuations in oxygen tension and nutrient supplies, which might contribute to the higher percentile LOH and GOH occurrences displayed by solid tumors compared to leukemias (Fig. 4b). Overall, the intimate relationship between defective DDR and cancer is clearly underlined by the numerous human genetic diseases that are associated with both DDR defects and predisposition to cancers including xeroderma pigmentosum, familial breast cancer, Bloom syndrome, MYH-associated polyposis, hereditary nonpolyposis colorectal cancer, etc. [59].

Accordingly, in the pre-oncogenesis phase, the weakening of cell cycle checkpoints caused by oncogene-induced DDR alterations can usher in mutations to initiate oncogenesis [46, 50–52]. In the post-oncogenesis phase, a defective DDR allows the continued accumulation of LOHs and GOHs, which can be self-amplifying insofar that some of the accumulated mutations can further alter DDR, to result in the wide landscape of mutations including LOHs, GOHs, CNVs, indels and chromosomal instabilities that characterize cancers, thus meeting fully the mutations needed for post-oncogenesis alterations which may be expected to outnumber those required for oncogenesis. Table 2 illustrates some of the LOH and GOH mutations in tumor suppressor and other cancer related genes potentially arising from interhomolog recombination enabled by a defective DDR that could be utilized by various cancers.

**Fig. 8:**
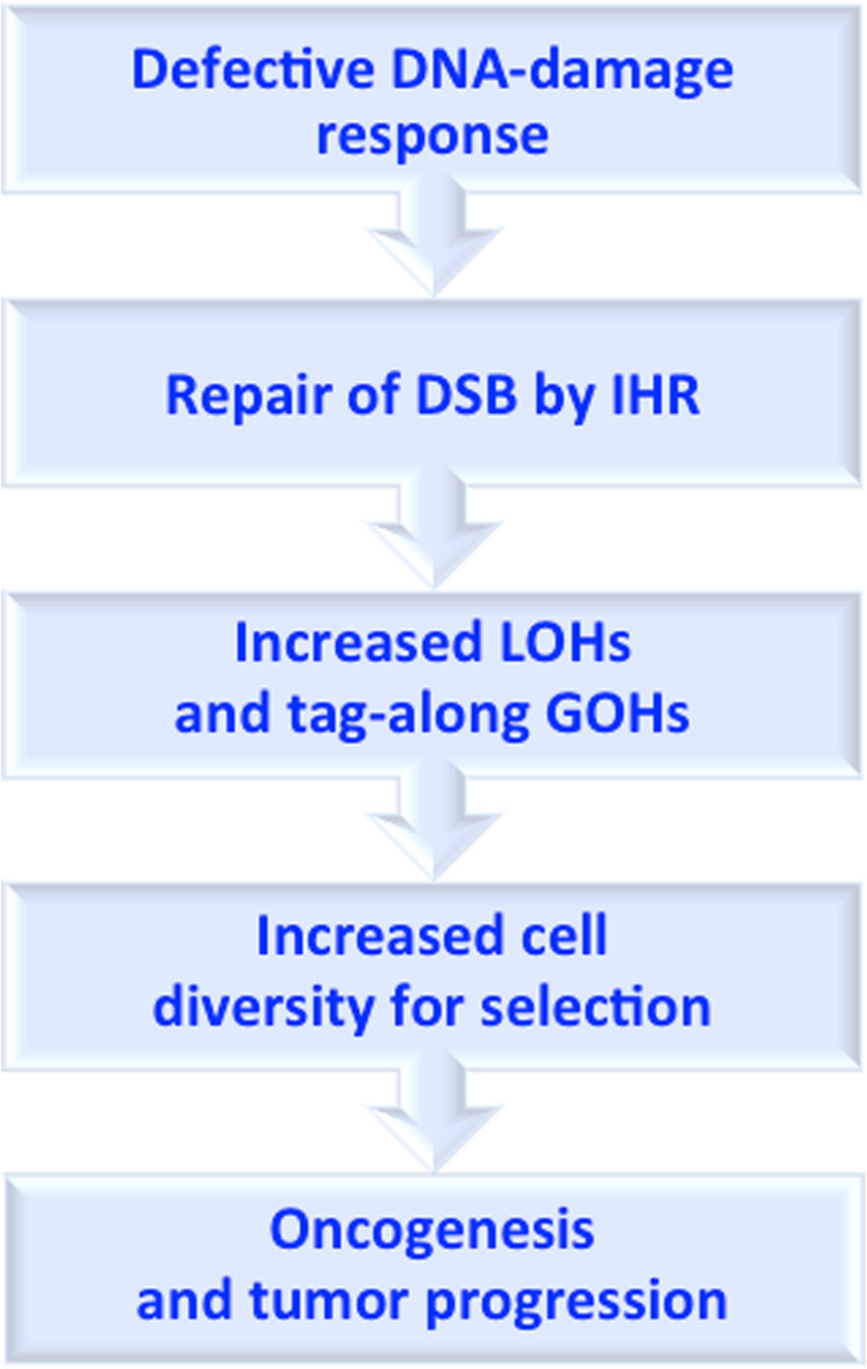
A unified mechanism for the generation of mutations underlying oncogenesis and tumor progression based on defective DNA-damage response and the resultant prevalence of double-strand break (DSB) repair by interhomolog recombination (IHR)

In conclusion, analysis of cancer LOHs and GOHs in the present study has revealed evidence for the occurrence of LOHs and tag-along GOHs in cancers brought about by repair of DSBs through interhomolog recombination under conditions of relaxed cell cycle checkpoints due to a defective DDR. On this basis, cancer may be regarded foremost as a disease of the DNA-damage response, where the mutator phenotype arising from DDR derangement provides a unified mechanism for generating interhomolog recombination-induced mutations (Fig. 8) to drive the initiation, development and aggressiveness of the neoplastic state from its oncogenic beginning to its terminal stages of unconstrained growth and proliferation.

## Availability of supporting data

All supporting data are included as additional files.

## Additional files

Additional file 1: Table S2. Different types of point mutations in cancer samples grouped by genotypes. Additional file 2:
**Methods.**
Additional file 3: Table S1. Summary of tumor-control pairs analyzed. Additional file 4: Table S3. Numbers of genotypes and SNV mutations on different chromosomes in various cancer samples. Additional file 5: Table S6 Complete list of CNV sites. Additional file 6: Figure S1. Chromosomal distributions of LOHs, GOHs and CNVs in cancer samples. Symbols representing the different types of mutations are given in legend of Fig. 1. Additional file 7: Table S4. Complete list of LOH sites and their CNV status. Additional file 8: Table S5. Complete list of GOH sites and their CNV status. Additional file 9: Table S7. LOH- and GOH-bearing genes present in TSGene, NCG4.0 and Ensembl databases. Additional file 10: Table S8. Proteins identified by CIPHER as high-risk for the solid tumor group. The gene ranks indicated in % for the high-risk genes in the four different types of cancers were predicted by CIPHER. Covered length refers to the average length of gene sequence captured by the 30 AluScans analyzed in Table 2. Additional file 11: Table S9. LOH fragment lengths. Additional file 12: Figure S2. Preferences for reference alleles and transitional changes displayed by LOH mutations in thirty cancer samples analyzed by AluScan. Symbols representing the different types of LOHs are given in legend of Fig. 3.

## Abbreviations

BWA: Burrows-Wheeler Aligner
CGH: Comparative genome hybridization
CNV: Copy number variation
DDR: DNA damage response
DSB: Double strand break
FS: Fisher’s exact test to detect strand bias
GATK: Genome Analysis Tool-Kit
GOH: Gain of heterozygosity
HR: Homologous recombination
ISR: Inter-sister chromatid recombination
IHR: Inter-homolog recombination
LOH: Loss of heterozygosity
QD: Quality by Depth
R_GOH-M_: Percentile mutation of MM residues in the form of GOH
R_GOH-m_: Percentile mutation of mm residues in the form of GOH
R_LOH_: Percentile mutation of Mm residues in the form of LOH
R_Mm_: Percentile mutation of Mm residues
R_mm_: Percentile mutation of mm residues
SAM: Sequence Alignment/Map
SNP: Single nucleotide polymorphism
WGS: Whole genome sequencing
