# Supplementary material for: Massive interstitial copy-neutral loss-of-heterozygosity as evidence for cancer being a disease of the DNA-damage response
Source: BMC Med Genomics. 2015 Jul 25;8:42. doi: 10.1186/s12920-015-0104-2 (PMC4515014; doi:10.1186/s12920-015-0104-2)
Supplement: Additional file 2: — Methods. [file 12920_2015_104_MOESM2_ESM.docx]

# SUPPLEMENTARY METHODS

1. **BWA alignment (bwa-0.6.1)**
   1. **Align reads**

bwa index hg19.fa

bwa aln -I -t [int] hg19.fa read_1.fq > read_1.sai

bwa aln -I -t [int] hg19.fa read_2.fq > read_2.sai

-I is added when Illumina version is 1.3 or 1.5. For version 1.8, -I is omitted.

-t [int] : [int] = number of thread

read: This is the name or ID of the sequence and must be the same as the sample ID

- 1. **Pair up alignment using BWA and sort results using SAMTools (SAMTools-0.1.19)**

bwa sampe hg19 read_1.sai read_2.sai read_1.fq read_2.fq | samtools view –bT hg19.fa -o read.bam -

samtools sort read.bam read.sorted

- 1. **Performance of primary statistics**

samtools flagstat read.sorted.bam

This command can determine the quality of the sequence. 80% mapped rate is satisfactory.

- 1. **Add header and ReadGroup**

### Create header file

echo -e “@RG\tID:read\tSM:read\tLB:ga\tPL:Illumina” > read.txt

### Add header and ReadGroup into BAM file

samtools view –h read.sorted.bam | cat read.txt - | awk ‘{if(substr($1,1,1)==”@”)print; else printf “%s\tRG:Z:read\n”,$0;}’ | samtools view –bS - > read.gatkready.bam

At “%s\tRG:Z:read\n”, the ‘read’ should be the same ID as in the header file.

### Remove duplicates

samtools rmdup read.recal.realigner.bam read.rmdup.bam

Index the file bam file before proceeding to GATK using the following command

samtools index read.rmdup.gatkready.bam

- 1. **Add or Replace ReadGroups by Picard**

Java -jar picard-tools-1.109/picard-tools-1.109/AddOrReplaceReadGroups.jar INPUT=read.rmdup.gatkready.bam OUTPUT=read_changedRG.gatkready.bam CREATE_INDEX=TRUE SORT_ORDER=coordinate RGID=Tumor RGLB=ga RGPU=Tumor RGPL=ILLUMINA RGSM=Tumor VALIDATION_STRINGENCY=LENIENT

# Alternate option, we can also use SAMTools for the same purpose to change ReadGroup.

1. **Variant calling by GATK (for GATK version 2.1-8)**
   1. **GATK recalibration and realignment**

java -Xmx4g -jar ./app/ GenomeAnalysisTKLite-2.1-8-gbb7f038/ GenomeAnalysisTKLite.jar -R hg19.fa -T BaseRecalibrator -knownSites dbsnp_137.hg19.vcf --disable_indel_quals -I read_changedRG.gatkready.bam -o read.recaldata.grp

java -Xmx4g -jar GATK.jar -R hg19.fa -T PrintReads -BQSR read.recaldata.grp -I read._changedRG.gatkready.bam -o read.recal.bam

java -Xmx4g -jar GATK.jar -R hg19.fa -T RealignerTargetCreator --known dbsnp_137.hg19.vcf -I read.recal.bam -o read.intervals

java -Xmx4g -jar GATK.jar -R hg19.fa -T IndelRealigner -known dbsnp_137.hg19.vcf -targetIntervals read.intervals -I read.recal.bam -o read.recal.realigner.bam

- 1. **Obtain variant sites by GATK**

java -jar GATK.jar -nt [Int] -R hg19.fa -D dbsnp_137.hg19.vcf -l INFO -T UnifiedGenotyper -I read1.recal.realigner.bam -I read2.recal.realigner.bam … -o read12.raw.snp.vcf

Then make an interval file named by using “.intervals” in tail

awk ‘{if($0!~/#/)print;}’ read12.raw.snp.vcf | awk ‘{if($0!~/\.\/\./)print;}’ - | awk ‘{printf ”%s:%s-%s\n”,$1,$2,$2;}’ - > read12.intervals

Then make a shared file of paired samples:

awk ‘{if($0!~/#/)print;}’ read.raw.snp.vcf | awk ‘{if($0!~/\.\/\./)print;}’ - > read12.shared.snp.vcf

- 1. **Run GATK again to call genotypes for control and test samples**

java -jar GATK.jar –nt [Int] -R hg19.fa -D dbsnp_137.hg19.vcf -l INFO -T UnifiedGenotyper -A BaseCounts -out_mode EMIT_ALL_SITES -L read12.intervals -I read1. recal.realigner.bam -o read1.shared.raw.snp.vcf

java -jar GATK.jar –nt [Int] -R hg19.fa -D dbsnp_137.hg19.vcf -l INFO -T UnifiedGenotyper -A BaseCounts -out_mode EMIT_ALL_SITES -L read12.intervals -I read2. recal.realigner.bam -o read2.shared.raw.snp.vcf

1. **Mutation analysis**
   1. **Pairing of two variant calling results**

perl multisample_analysis.pl -SN (sample number) -SEX (subject sex) -L read12.shared.snp.vcf -out read12.combined.raw.snp.txt read1.shared.raw.snp.vcf(input1) read2.shared.raw.snp.vcf(input2)

- 1. **Filtration conditions**

perl multisample_filter.pl -SN (sample number) -SEX (subject sex) -in read12.combined.raw.snp.txt -out read12.flt.snp.txt -DP 8 –AF1 0 –AF2 0.35 –AF3 0.65 -AF4 1 -QD1 4 -QD2 20 –SB -0.01

Note:

#AF: minor allele frequency. We call minor allele which is different from reference allele in human reference genome (hg19).

#AF1: minor allele frequency value for homozygous reference sites is set to be 0.

#AF2, AF3: To call heterozygous site we fix minor allele frequency between 0.35 and 0.65, so AF2 and AF3 values are fixed to 0.35 and 0.65 respectively.

#AF4: we fix minor allele frequency 1 to call homozygous non-reference sites.

##QD stands for quality by depth.

#QD1: quality by depth QD1 value for heterozygous site is fixed to ≥4.

#QD2: QD2 value should be ≥20 to call homozygous non-reference sites.

##DP: DP stands for depth.

#DP: depth value is set to be ≥8 for our genotype calling.

#SB: strand bias value should be lower than -0.01.

- 1. **Mutation calling**

**For mutation calling from paired samples**

awk '{if($9!=$15)print;}' read12.flt.snp.txt > read12.flt.snv.txt

**LOH and GOH mutation calling**

perl somatic_type.pl read12.flt.snv.txt - | grep "GOH" - > ./read12_snv.GOH.txt

perl somatic_type.pl read12.flt.snv.txt - | grep "LOH" - > ./read12_snv.LOH.txt

1. **Mutation pattern statistics**
   1. **Separate mutation sites based on reference alleles**

perl RF_A_separator.pl read12.flt.snp.txt > read12_A_Sites_result.txt

perl RF_G_separator.pl read12.flt.snp.txt > read12_G_Sites_result.txt

perl RF_T_separator.pl read12.flt.snp.txt > read12_T_Sites_result.txt

perl RF_C_separator.pl read12.flt.snp.txt > read12_C_Sites_result.txt

#we combine the four commands above in one single file “RF_Separater.pl” to run together

perl RF_Separator.pl

#read12_A_Sites_result.txt shows a mutation list when reference allele is A

#read12_G_Sites_result.txt shows a list when reference allele is G

#read12_T_Sites_result.txt shows a list when reference allele is T

#read12_C_Sites_result.txt shows a list when reference allele is C

- 1. **Mutation count based on reference alleles**

perl Mutation_count.pl read12_A_Sites_result.txt > read12_A_Mutation_count.txt

perl Mutation_count.pl read12_G_Sites_result.txt > read12_G_Mutation_count.txt

perl Mutation_count.pl read12_T_Sites_result.txt > read12_T_Mutation_count.txt

perl Mutation_count.pl read12_C_Sites_result.txt > read12_C_Mutation_count.txt

# we combine the four commands above in one single file “Mutation_Count.pl” to run together

# read12_A_Mutation_count.txt shows a mutation list when reference allele is A

# read12_G_Mutation_count.txt shows a list when reference allele is G

# read12_T_Mutation_count.txt shows a list when reference allele is T

# read12_C_Mutation_count.txt shows a list when reference allele is C

# perl Mutation_Count.pl

* All mutated and non mutated site details for homozygous non-reference and heterozygous calling are available in read12.flt.snp.txt. For a particular number or specific sites validation follow read12.flt.snp.txt.

## Count number of homozygous non-reference and heterozygous sites captured in control sample

perl Alt_Het_count.pl read12.flt.snp.txt > read12_Alt_Het_sites.txt

### Count homozygous reference sites

java –jar GenomeAnalysisTKLite.jar –nt 8 -T UnifiedGenotyper -R hg19.fa -D dbsnp_137.hg19.vcf -I read1.realigner.bam -stand_call_conf 50 -stand_emit_conf 10 -o read1.raw.ALL.vcf -A BaseCounts -out_mode EMIT_ALL_CONFIDENT_SITES

### Filter reference sites based on filter depth >= 8

awk '{if($0!~/SB/) print;}' read1.raw.ALL.vcf | awk -F ':' '{print$1"\t"$2"\t"$3"\t"$4"\t" ;}' - | awk '{if($12>=8) print;}' - > read1_RF_DP8.txt

#similarly make file read2_RF_DP8.txt

#Take intersection sites of two samples, read1_RF_DP8.txt and read2_RF_DP8.txt and make file, we use Varscan tools for intersection task.

java –jar VarScan.v2.3.4.jar compare read1_RF_DP8.txt read2_RF_DP8.txt intersect read12_RF_intersect.txt

### Calculate Number of non-mutated reference sites captured based on reference alleles

perl bp_count.pl read12_RF_intersect.txt > Total_non_mutated_RF_count.txt

# Total_non_mutated_RF_count.txt file contains how many sites of reference allele A, G, T and C remain as non-mutated

# Control-FREEC CNV Calling:

## Preparation of file for intersected coverage between case and control

samtools depth -q 0 -Q 0 -b in.bed read.rmdup.bam | awk '$3 >= 8' - |

perl depth2bed.pl - read1.cov8.bed read1.cov8.stat

#-b: list of positions or region.

#-q: base quality threshold

#-Q: mapping quality threshold

This will create a BED file for read1 with depths >= 8

Do the same for read2, and find out the intersection by using intersectBed in bedtools

./BEDTools-Version-2.16.2/bin/intersectBed -a read1.cov8.bed -b read2.cov8.bed > read12.intersect.bed

## Make configure file and run Control-FREEC

We make a FREEC CNV config file with name Config_read12.txt with all parameters, and run the command below

. /freec -conf Config_read12.txt

1. **AluScanCNV workflow**

## Prepare files for scaling

### perl bin.pl 5000 > 5k.bin

# Input: binsize

# Output: 5k.bin

# the file “hg19.len” should be in the current directory.

# Function: split genome into different windows

# the chromosome number will have "chr" in prefix; no header in the output; chrX and chrY represent chromosome X and chromosome Y respectively**.**

### perl bin2pos.pl 5k.bin > 5k.pos

# Input: bin file

# Output: pos file

# Function: transform bin format to pos format; the pos file will be used to calculate GC content

# the chromosome number will NOT have "chr" in front; there is a header in the output; 23 and 24 represent chromosome X and chromosome Y respectively.

perl factor.pl 5k.pos 500k.bin > F500k.factor

# Input: 5k.pos, bin file with larger binsize that is integral multiples of 5k

# Output: factor file

# Function: mapping 5k.bin to different binsize in 500k.bin

## Prepare information files

To make reference file and sample file, references_ID.txt and samples_ID.txt

# Format is:

TTG160B F

TTG176B M

LG3B F

TTG153B M

...

# the first column is ID, the second column is gender

# prepare sample information table in a format like:

Type Disease Sample Gender

cancer_tissue glioma Glioma.056T F

cancer_tissue leukemia Leukemia.Sample3T11 M

cancer_blood leukemia Leukemia.Sample3N12 M

cancer_blood glioma Glioma.216B F

cancer_tissue glioma Glioma.216T F

...

# the first column is tissue feature, the second column is disease type, the third column is sample ID, and the fourth column is gender

## Generate main table for AluScanCNV calling

# apply SAMTools and BEDTools to make the coverage files (.doc)

samtools rmdup input.bam - | bam2bed - > output.bed

coverageBed -a output.bed -b 5k.bin > output.5k.doc

# put all the doc files into an inputfileList like:

TTG160B.5k.doc

TTG160T.5k.doc

TTG176B.5k.doc

TTG176T.5k.doc

Gastric1B.5k.doc

Gastric1T.5k.doc

...

Perl combine.pl inputfileList 5k Reads.5k.data

# Input: inputfileList, binsize

# Output: Reads.data

# combine all the coverage and binsize information to construct a matrix for following analysis

## Extended CNV and localized CNV calling

# please refer to demo.R

# input: Reads.5k.data, 5k.pos, 5k.bin, samples_ID.txt, references_ID.txt, All.samples.ID.txt, F500k.factor, 500k.pos, 500k.bin, hg19.fa

# Due to the upload limit, here we only upload a shortcut version of Reads.5k.data with its 100 rows at the front in Demo Input. If you need the complete version of Reads.5k.data, please contact us

1. **LOH and GOH analysis**

## Obtain information for each locus (LOH or GOH) from UCSC database

perl Loci_info.pl RepeatMasker.hg19 ucsc_hg19_ensGene.info read12_snv.LOH.txt read12_snv.LOH.info

perl Loci_info.pl RepeatMasker.hg19 ucsc_hg19_ensGene.info read12_snv.GOH.txt read12_snv.GOH.info

## Find relationship between LOH/GOH and purposed gene database (TSGene/NCG )

perl find_gene.pl read12_snv.LOH.info [database_file] read12_snv.LOH.info.tsg

[database_file]: tsg or ncg

## Find occurrence of LOH/GOH in gene databases from 30 AluScan data

We combine all 30 sample files of AluScan data. Please note that to run the following command, all the files should be in the current directory.

perl find_occurrence_in_Alu30.pl occurrence_Alu30_LOH_tsg.file LOH tsg > log_file

The ‘occurrence_Alu30_LOH_tsg.file’ is the output.

## Determine number of sites captured in genic regions

perl genesites_captured.pl samplefile Gene-list > total_sites_captured

# Gene-list file should have at least the three columns of Chromosome, GeneStart and GeneEnd.
