# Supplementary material for: Massive interstitial copy-neutral loss-of-heterozygosity as evidence for cancer being a disease of the DNA-damage response
Source: BMC Med Genomics. 2015 Jul 25;8:42. doi: 10.1186/s12920-015-0104-2 (PMC4515014; doi:10.1186/s12920-015-0104-2)
Supplement: Additional file 3: Table S1. — Summary of tumor-control pairs analyzed. [file 12920_2015_104_MOESM3_ESM.docx]

**Table S1. Summary of tumor-control pairs analyzed**

**Table S1A. Numbers of nucleotides analyzed and genotype frequencies**

| **Sample** ^a^ | **Nucleotide Positions Analyzed** (bp) | | | | **Genotype Frequency** (%) | | |
| --- | --- | --- | --- | --- | --- | --- | --- |
|  | **Total** | **MM** | **mm** | **Mm** | **MM** | **mm** (x 10^-2^) | **Mm** (x 10^-2^) |
| Gastric 1 | 16084161 | 16080699 | 1222 | 2240 | 99.978 | 0.760 | 1.139 |
| Gastric 2 | 24503645 | 24491876 | 5157 | 6612 | 99.952 | 2.105 | 2.698 |
| Gastric 3 | 24844283 | 24834488 | 5067 | 4728 | 99.961 | 2.040 | 1.903 |
| Gastric 4 | 18215424 | 18209732 | 2417 | 3275 | 99.969 | 1.327 | 1.798 |
| Gastric 5 | 16667874 | 16663684 | 989 | 3201 | 99.975 | 0.593 | 1.920 |
| Glioma 1 | 18923296 | 18916452 | 2339 | 4505 | 99.964 | 1.236 | 2.381 |
| Glioma 2 | 9840282 | 9836329 | 1680 | 2273 | 99.960 | 1.707 | 2.310 |
| Glioma 3 | 21079231 | 21070588 | 4158 | 4465 | 99.959 | 1.973 | 2.118 |
| Glioma 4 | 8887856 | 8883912 | 1953 | 1991 | 99.956 | 2.197 | 2.240 |
| Glioma 5 | 10489191 | 10485371 | 1863 | 1957 | 99.964 | 1.776 | 1.866 |
| Leukemia 1 | 15362821 | 15356651 | 2535 | 3635 | 99.960 | 1.650 | 2.366 |
| Leukemia 2 | 12995297 | 12989543 | 2370 | 3384 | 99.956 | 1.824 | 2.604 |
| Leukemia 3 | 21911433 | 21901408 | 3941 | 6084 | 99.954 | 1.799 | 2.777 |
| Leukemia 4 | 17746114 | 17741705 | 1263 | 3146 | 99.975 | 0.712 | 1.773 |
| Leukemia 5 | 20980047 | 20974726 | 1778 | 3543 | 99.975 | 0.847 | 1.689 |
| Liver 1 | 10849704 | 10847515 | 846 | 1343 | 99.980 | 0.780 | 1.237 |
| Liver 2 | 17729365 | 17722590 | 2323 | 4452 | 99.962 | 1.310 | 2.511 |
| Liver 3 | 12043041 | 12037621 | 2320 | 3100 | 99.955 | 1.926 | 2.574 |
| Liver 4 | 7932882 | 7930121 | 1078 | 1683 | 99.965 | 1.359 | 2.122 |
| Liver 5 | 11080478 | 11075760 | 2198 | 2520 | 99.957 | 1.984 | 2.274 |
| Lung 1 | 11936081 | 11930379 | 2335 | 3367 | 99.952 | 1.956 | 2.821 |
| Lung 2 | 12962455 | 12956800 | 3111 | 2544 | 99.956 | 2.400 | 1.963 |
| Lung 3 | 14230061 | 14224973 | 1947 | 3141 | 99.964 | 1.368 | 2.207 |
| Lung 4 | 12491386 | 12486989 | 1606 | 2791 | 99.965 | 1.286 | 2.223 |
| Lung 5 | 14461911 | 14454748 | 2975 | 4188 | 99.950 | 2.057 | 2.896 |
| Lung-Brain 1 | 21687628 | 21673375 | 5830 | 8423 | 99.934 | 2.688 | 3.884 |
| Lung-Brain 2 | 21684922 | 21672692 | 5274 | 6956 | 99.943 | 2.432 | 3.208 |
| Lung-Brain 3 | 17036555 | 17027704 | 3226 | 5625 | 99.948 | 1.894 | 3.302 |
| Lung-Brain 4 | 22791303 | 22779503 | 4298 | 7502 | 99.948 | 1.886 | 3.292 |
| Lung-Brain 5 | 21042223 | 21028897 | 5699 | 7627 | 99.937 | 2.708 | 3.625 |
| **Leukemia**  **Av. ± SD** | **17799142**  **±3743143** | **17792806**  **±3742125** | **2377**  **±1008** | **3958**  **±1202** | **99.964**  **±0.010** | **1.366**  **±0.542** | **2.242**  **±0.489** |
| **Solid tumor**  **Av. ± SD** | **15979809**  **±5100714** | **15972911**  **±5097764** | **2876**  **±1549** | **4020**  **±2041** | **99.958**  **±0.011** | **1.750**  **±0.571** | **2.420**  **±0.678** |
| **All sample**  **Av. ± SD** | **16283031**  **±4892819** | **16276227**  **±4890197** | **2793**  **±1470** | **4010**  **±1910** | **99.959**  **±0.011** | **1.686**  **±0.576** | **2.391**  **±0.647** |
| Lung-Liver (WGS) | 1422885430 | 1420364145 | 909429 | 1611856 | 99.823 | 6.391 | 11.328 |
| Liver (WGS) | 2601547317 | 2598744922 | 1243512 | 1558883 | 99.892 | 4.780 | 5.992 |

**Table S1B. Various forms of single nucleotide variations and genotype mutation rates**

| **Sample** ^a^ | **GOH-M** ^b^ | **GOH-m** ^b^ | **LOH** | **F_GOH-M_** ^c^ | **F_GOH-m_** ^c^ | **F_LOH_** ^c^ | **R_MM_** (10^-3^ ％) | R_mm_ (％) | **R_Mm_** (％) |
| --- | --- | --- | --- | --- | --- | --- | --- | --- | --- |
| Gastric 1 | 99 | 14 | 492 | 16.364 | 2.314 | 81.322 | 0.616 | 1.146 | 22.277 |
| Gastric 2 | 130 | 37 | 28 | 66.667 | 18.974 | 14.359 | 0.531 | 0.737 | 0.544 |
| Gastric 3 | 55 | 7 | 113 | 31.429 | 4.000 | 64.571 | 0.221 | 0.138 | 2.813 |
| Gastric4 | 54 | 10 | 84 | 36.486 | 6.757 | 56.757 | 0.297 | 0.414 | 2.809 |
| Gastric5 | 101 | 9 | 96 | 49.029 | 4.369 | 46.602 | 0.606 | 0.910 | 3.062 |
| Glioma1 | 32 | 2 | 64 | 32.653 | 2.041 | 65.306 | 0.169 | 0.086 | 1.421 |
| Glioma2 | 46 | 5 | 210 | 17.624 | 1.916 | 80.460 | 0.468 | 0.298 | 13.154 |
| Glioma3 | 647 | 163 | 996 | 35.825 | 9.025 | 55.150 | 3.166 | 4.257 | 24.009 |
| Glioma4 | 644 | 3 | 85 | 87.978 | 0.410 | 11.612 | 7.294 | 0.154 | 4.671 |
| Glioma5 | 493 | 180 | 512 | 41.603 | 15.190 | 43.207 | 4.750 | 10.574 | 26.622 |
| Leukemia 1 | 14 | 1 | 10 | 56.000 | 4.000 | 40.000 | 0.091 | 0.039 | 0.275 |
| Leukemia 2 | 11 | 0 | 9 | 55.000 | 0.000 | 45.000 | 0.085 | 0.000 | 0.296 |
| Leukemia3 | 13 | 4 | 20 | 35.135 | 10.811 | 54.054 | 0.059 | 0.101 | 0.427 |
| Leukemia 4 | 21 | 3 | 32 | 37.500 | 5.357 | 57.143 | 0.118 | 0.238 | 1.207 |
| Leukemia 5 | 51 | 12 | 42 | 48.571 | 11.429 | 40.000 | 0.243 | 0.675 | 1.383 |
| Liver 1 | 417 | 87 | 359 | 48.320 | 10.081 | 41.599 | 3.881 | 10.520 | 27.104 |
| Liver 2 | 127 | 30 | 567 | 17.541 | 4.144 | 78.315 | 0.717 | 1.421 | 13.814 |
| Liver 3 | 71 | 20 | 162 | 28.063 | 7.905 | 64.032 | 0.590 | 0.862 | 6.387 |
| Liver 4 | 47 | 15 | 271 | 14.114 | 4.505 | 81.381 | 0.605 | 1.763 | 16.162 |
| Liver 5 | 39 | 5 | 166 | 18.571 | 2.381 | 79.048 | 0.352 | 0.227 | 7.302 |
| Lung 1 | 31 | 21 | 233 | 10.877 | 7.368 | 81.754 | 0.260 | 0.899 | 6.980 |
| Lung 2 | 318 | 41 | 291 | 48.923 | 6.308 | 44.769 | 2.454 | 1.479 | 11.635 |
| Lung 3 | 238 | 63 | 204 | 47.129 | 12.475 | 40.396 | 1.680 | 3.236 | 7.291 |
| Lung 4 | 351 | 103 | 151 | 58.016 | 17.025 | 24.959 | 2.811 | 6.476 | 5.661 |
| Lung 5 | 1537 | 454 | 2017 | 38.348 | 11.327 | 50.324 | 11.609 | 20.134 | 49.690 |
| Lung-Brain 1 | 175 | 33 | 155 | 48.209 | 9.091 | 42.700 | 0.807 | 0.583 | 2.125 |
| Lung-Brain 2 | 183 | 26 | 461 | 27.313 | 3.881 | 68.806 | 0.858 | 0.512 | 7.346 |
| Lung-Brain 3 | 96 | 38 | 63 | 48.731 | 19.289 | 31.980 | 0.564 | 1.209 | 1.707 |
| Lung-Brain 4 | 209 | 44 | 351 | 34.603 | 7.285 | 58.112 | 0.931 | 1.024 | 5.052 |
| Lung-Brain 5 | 111 | 8 | 71 | 58.421 | 4.211 | 37.368 | 0.528 | 0.158 | 1.285 |
| **Leukemia**  **Av. ± SD** | **22**  **±16** | **4**  **±4** | **22**  **±14** | **46.441**  **±9.707** | **6.319**  **±4.809** | **47.239**  **±7.974** | **0.119**  **±0.072** | **0.211**  **±0.275** | **0.718**  **±0.534** |
| **Solid Tumor**  **Av. ± SD** | **250**  **±324** | **56**  **±95** | **328**  **±413** | **38.513**  **±18.352** | **7.691**  **±5.413** | **53.796**  **±20.896** | **1.871**  **±2.666** | **2.769**  **±4.663** | **10.837**  **±11.503** |
| **All sample**  **Av. ± SD** | **212**  **±307** | **47**  **±88** | **277**  **±393** | **39.835**  **±17.342** | **7.462**  **±5.264** | **52.703**  **±19.398** | **1.579**  **±2.515** | **2.342**  **±4.352** | **9.150**  **±11.147** |
| Lung-Liver (WGS) | 691 | 71 | 2498 | 21.196 | 2.178 | 76.626 | 0.049 | 0.008 | 0.167 |
| Liver (WGS) | 4891 | 65 | 1883 | 71.516 | 0.950 | 27.533 | 0.190 | 0.016 | 0.144 |

^a^ 30 samples were analyzed by AluScan, and two by WGS^7,8^.

^b^ GOH-M: gain of heterozygosity from MM in control to Mm in tumor; GOH-m: gain of heterozygosity from mm in control to Mm in tumor.

^c^ F_GOH-M_: fraction of single nucleotide variations in the form of GOH-M; F_GOH-m_: fraction of single nucleotide variations in the form of GOH-m; F_LOH_: fraction of single nucleotide variations in the form of LOH.
