# Supplementary figures and images for: Massive interstitial copy-neutral loss-of-heterozygosity as evidence for cancer being a disease of the DNA-damage response
Source: BMC Med Genomics. 2015 Jul 25;8:42. doi: 10.1186/s12920-015-0104-2 (PMC4515014; doi:10.1186/s12920-015-0104-2)

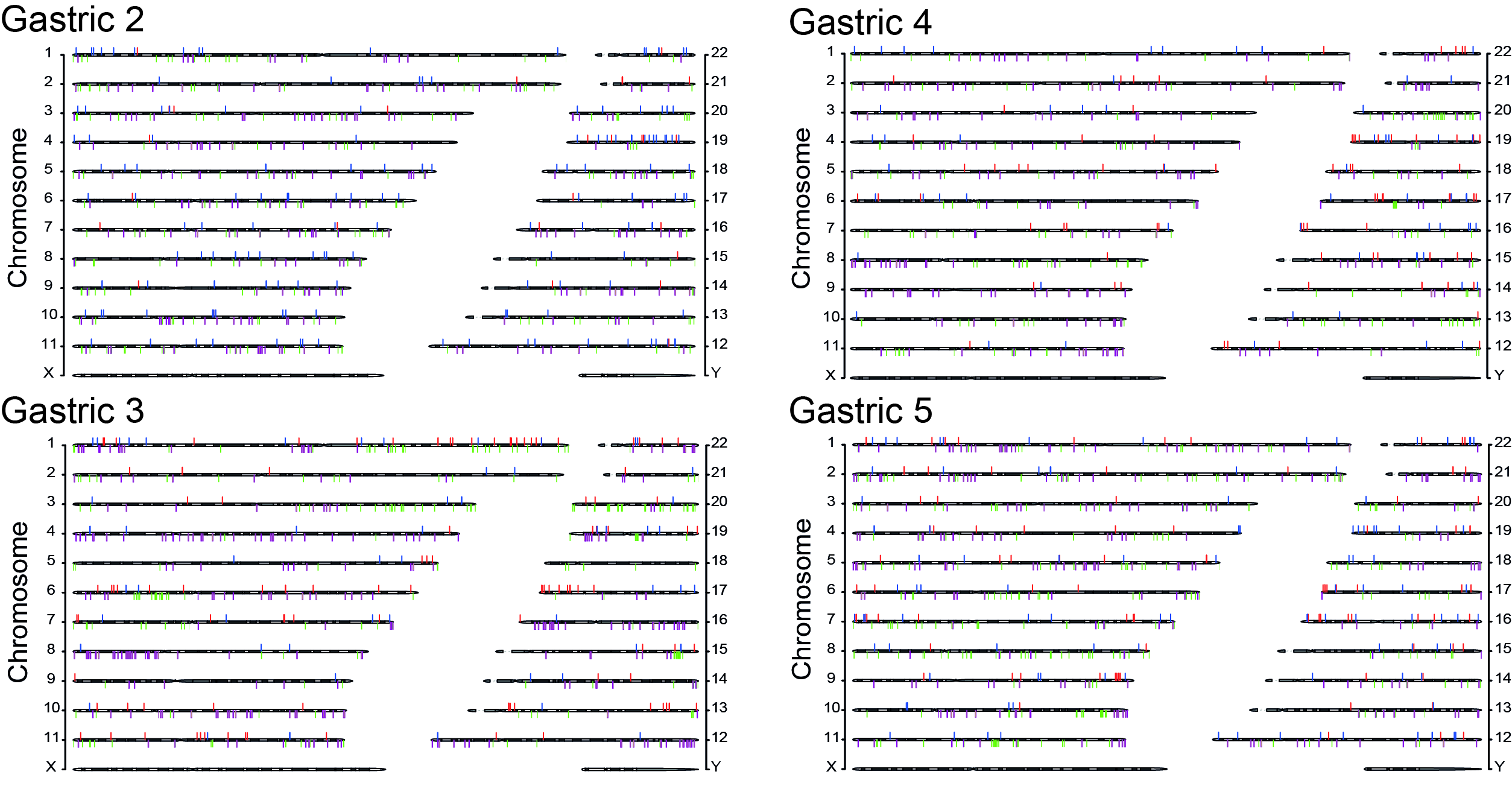

Supplement: Additional file 6: Figure S1. — Chromosomal distributions of LOHs, GOHs and CNVs in cancer samples. Symbols representing the different types of mutations are given in legend of Fig. 1. [file 12920_2015_104_MOESM6_ESM.zip › Figure S1/Figure S1A Gastric.tiff]

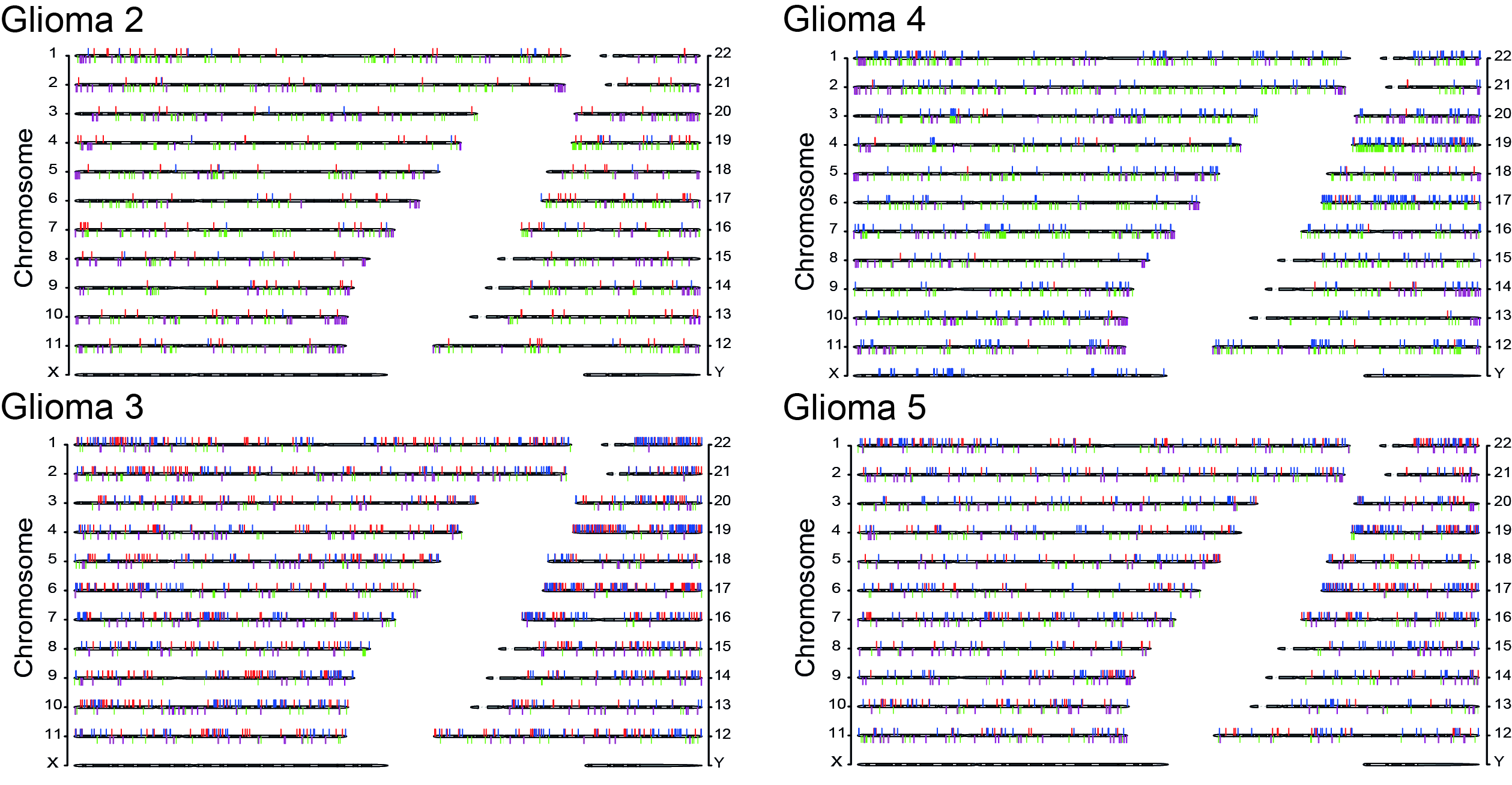

Supplement: Additional file 6: Figure S1. — Chromosomal distributions of LOHs, GOHs and CNVs in cancer samples. Symbols representing the different types of mutations are given in legend of Fig. 1. [file 12920_2015_104_MOESM6_ESM.zip › Figure S1/Figure S1B Glioma.tiff]

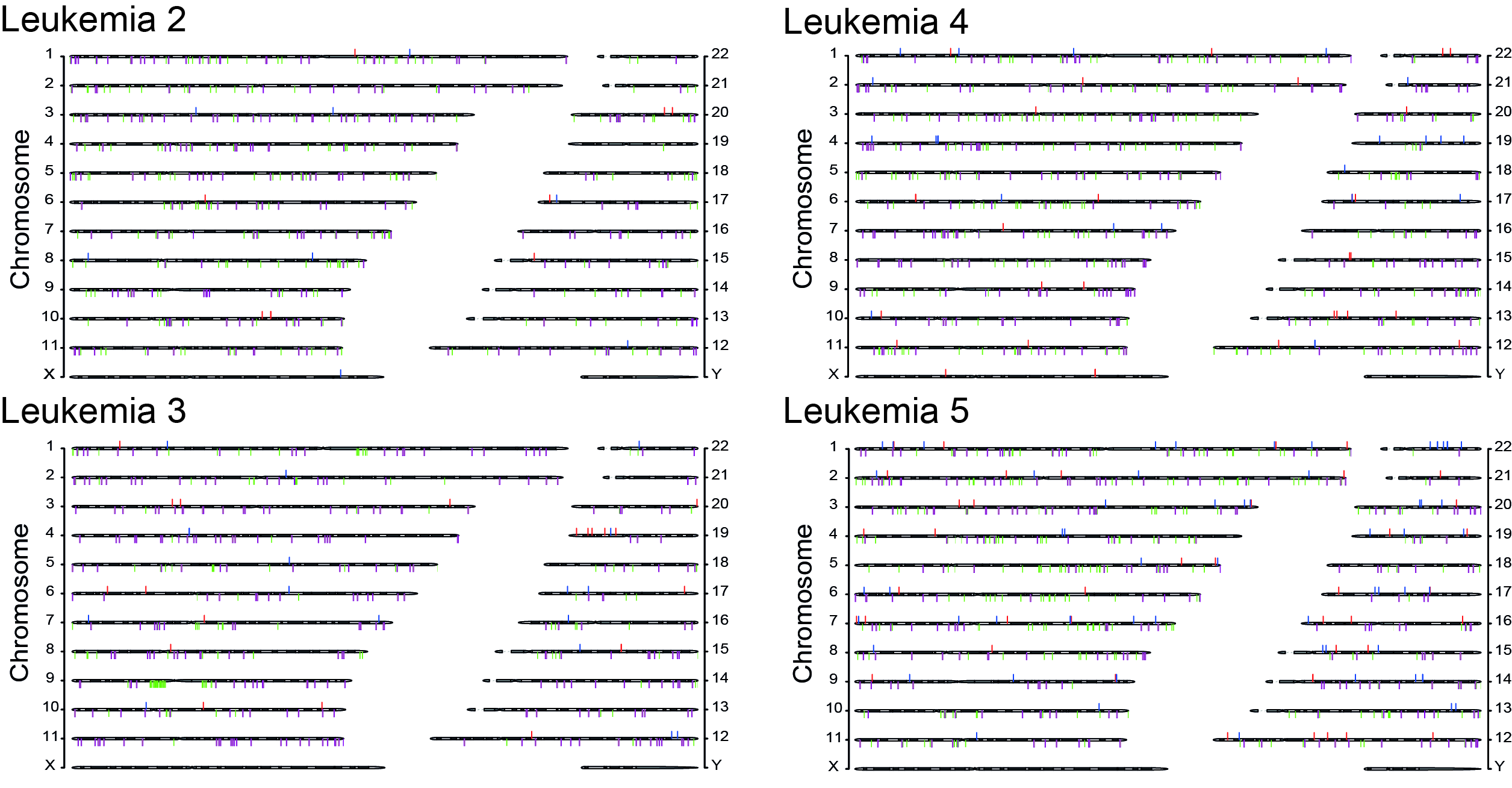

Supplement: Additional file 6: Figure S1. — Chromosomal distributions of LOHs, GOHs and CNVs in cancer samples. Symbols representing the different types of mutations are given in legend of Fig. 1. [file 12920_2015_104_MOESM6_ESM.zip › Figure S1/Figure S1C Leukemia.tiff]

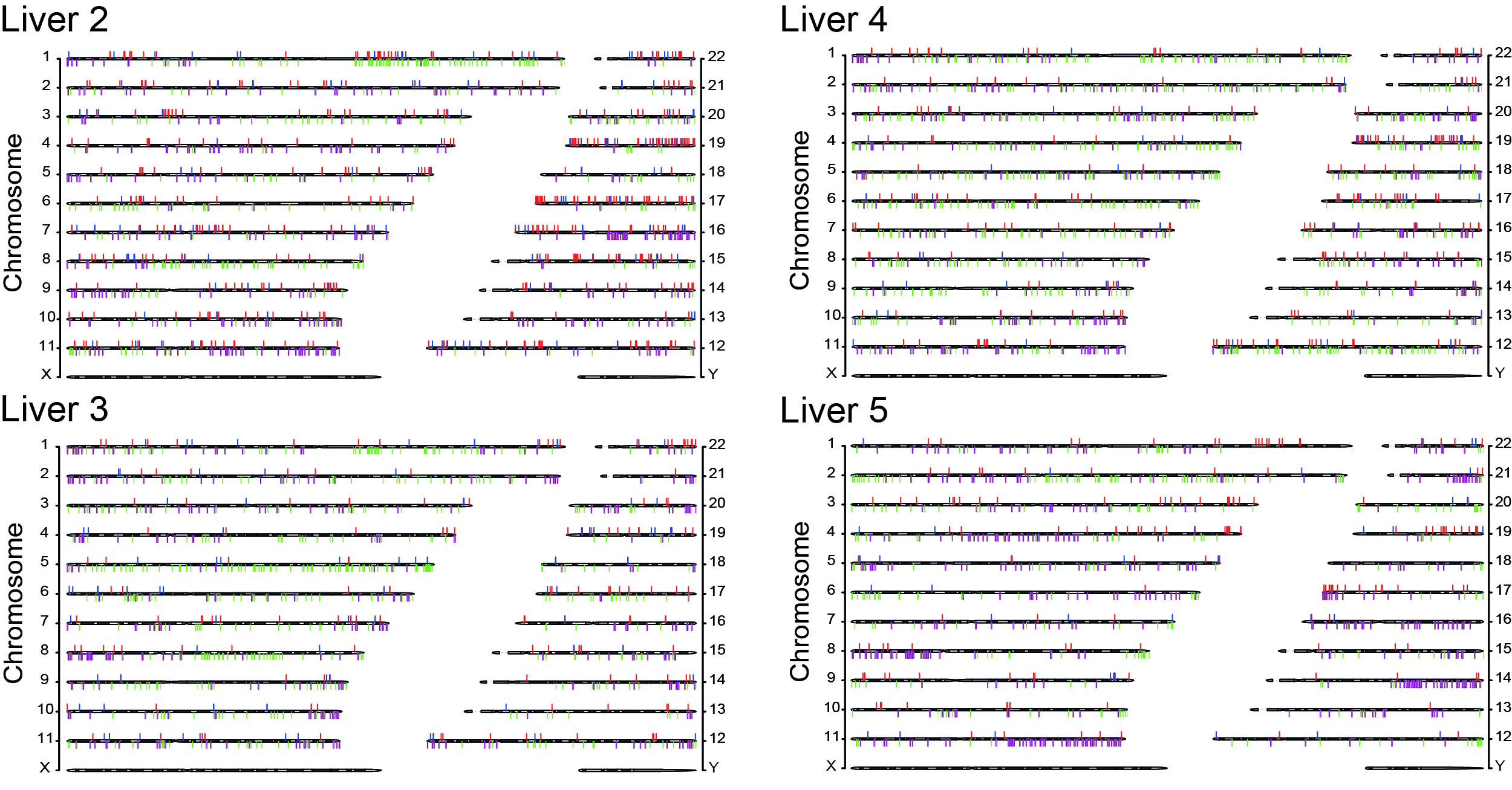

Supplement: Additional file 6: Figure S1. — Chromosomal distributions of LOHs, GOHs and CNVs in cancer samples. Symbols representing the different types of mutations are given in legend of Fig. 1. [file 12920_2015_104_MOESM6_ESM.zip › Figure S1/Figure S1D Liver.tiff]

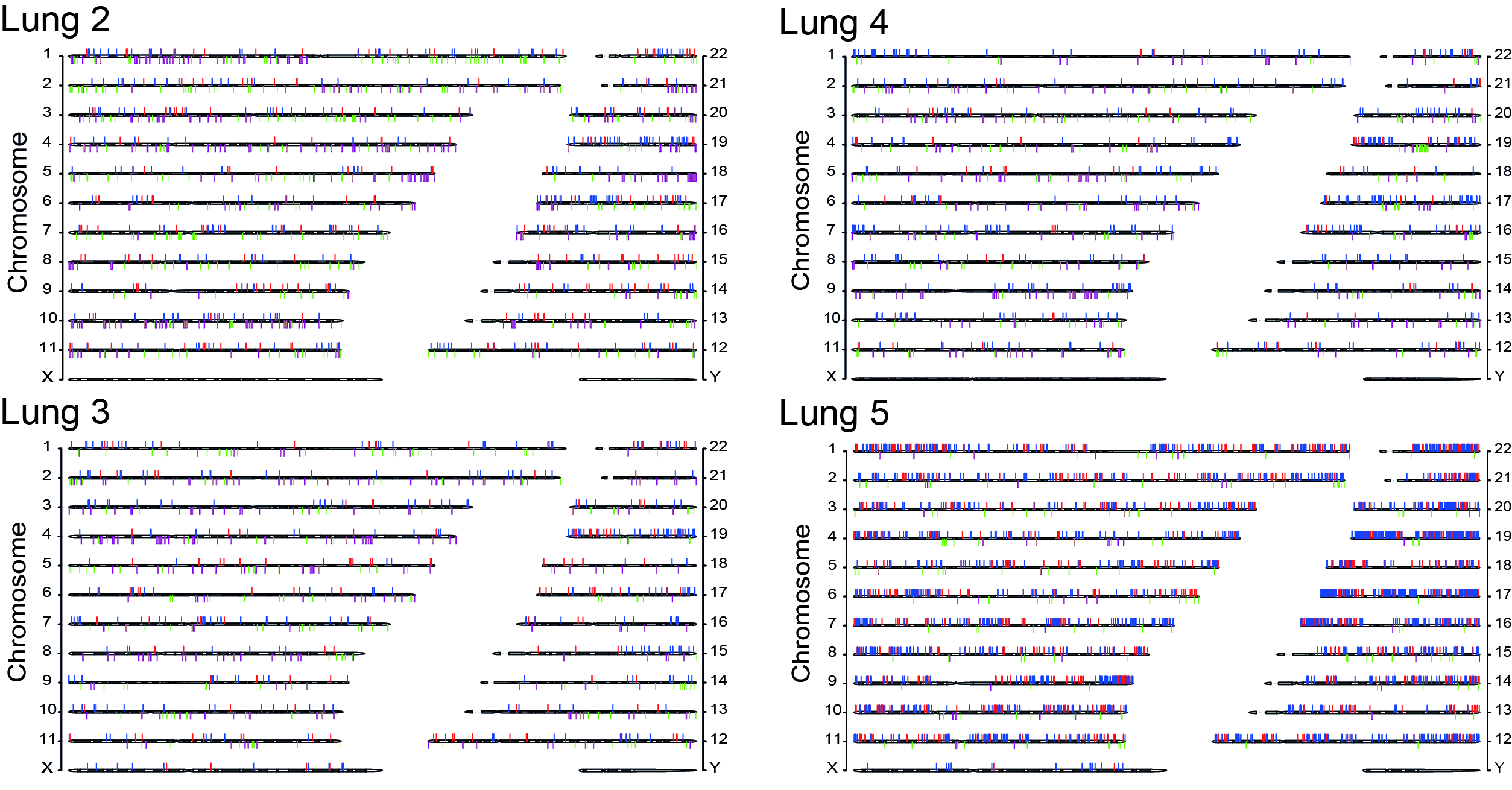

Supplement: Additional file 6: Figure S1. — Chromosomal distributions of LOHs, GOHs and CNVs in cancer samples. Symbols representing the different types of mutations are given in legend of Fig. 1. [file 12920_2015_104_MOESM6_ESM.zip › Figure S1/Figure S1E Lung.tiff]

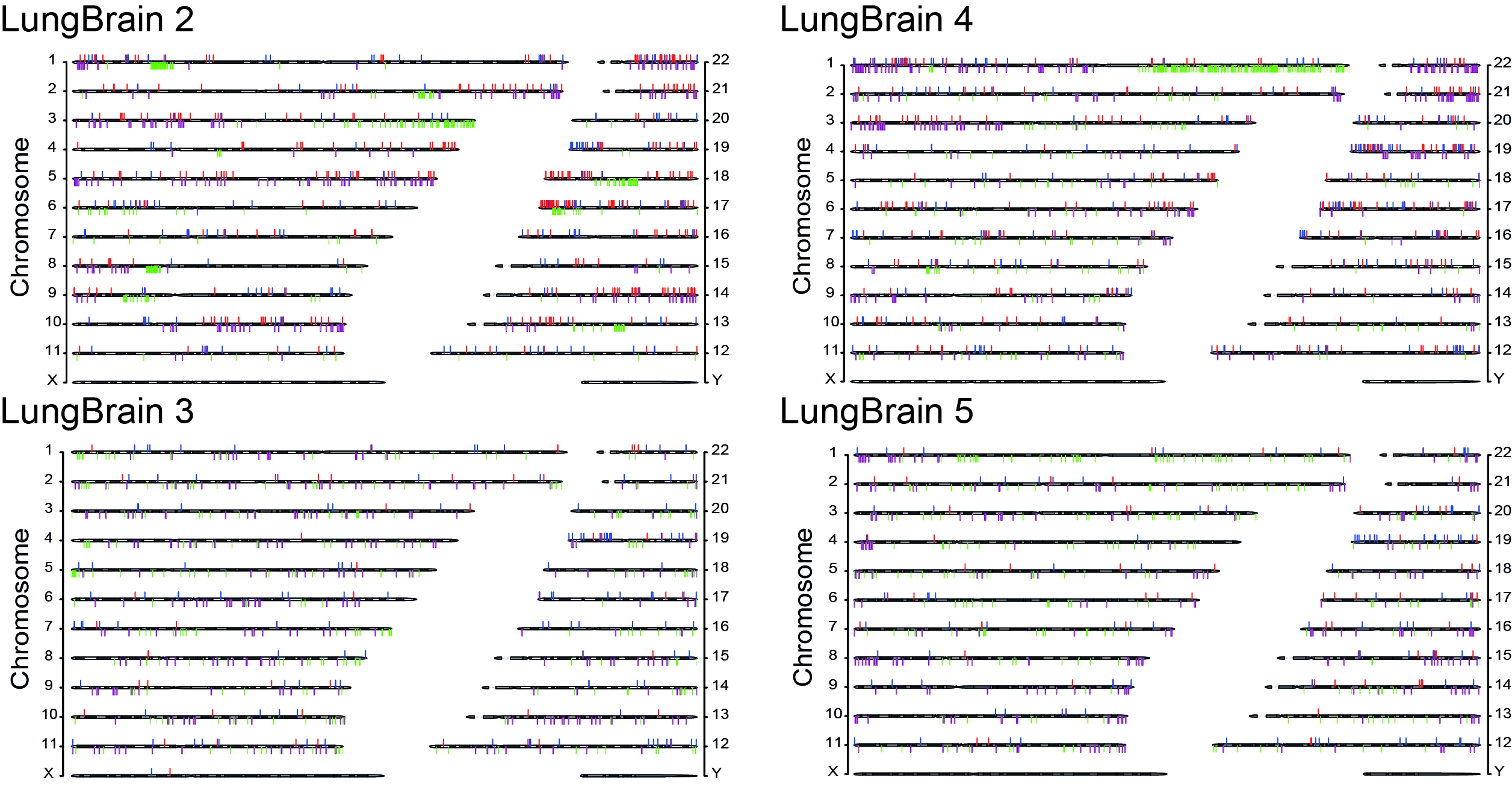

Supplement: Additional file 6: Figure S1. — Chromosomal distributions of LOHs, GOHs and CNVs in cancer samples. Symbols representing the different types of mutations are given in legend of Fig. 1. [file 12920_2015_104_MOESM6_ESM.zip › Figure S1/Figure S1F LungBrain.tiff]

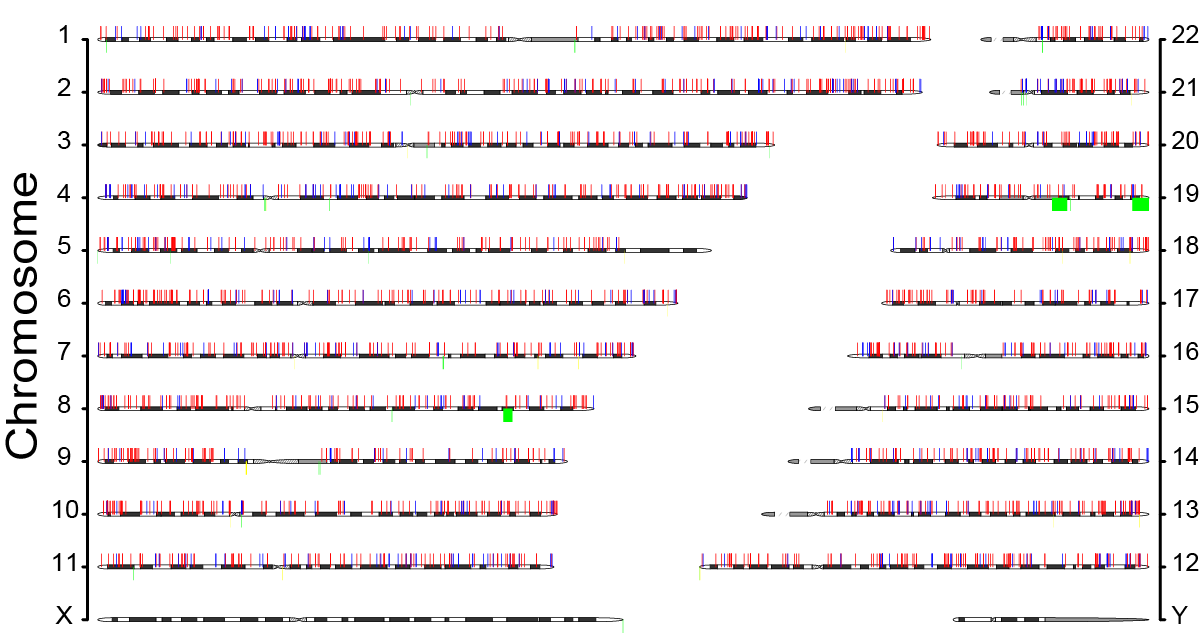

Supplement: Additional file 6: Figure S1. — Chromosomal distributions of LOHs, GOHs and CNVs in cancer samples. Symbols representing the different types of mutations are given in legend of Fig. 1. [file 12920_2015_104_MOESM6_ESM.zip › Figure S1/Figure S1G Lung-to-Liver (WGS).tif]

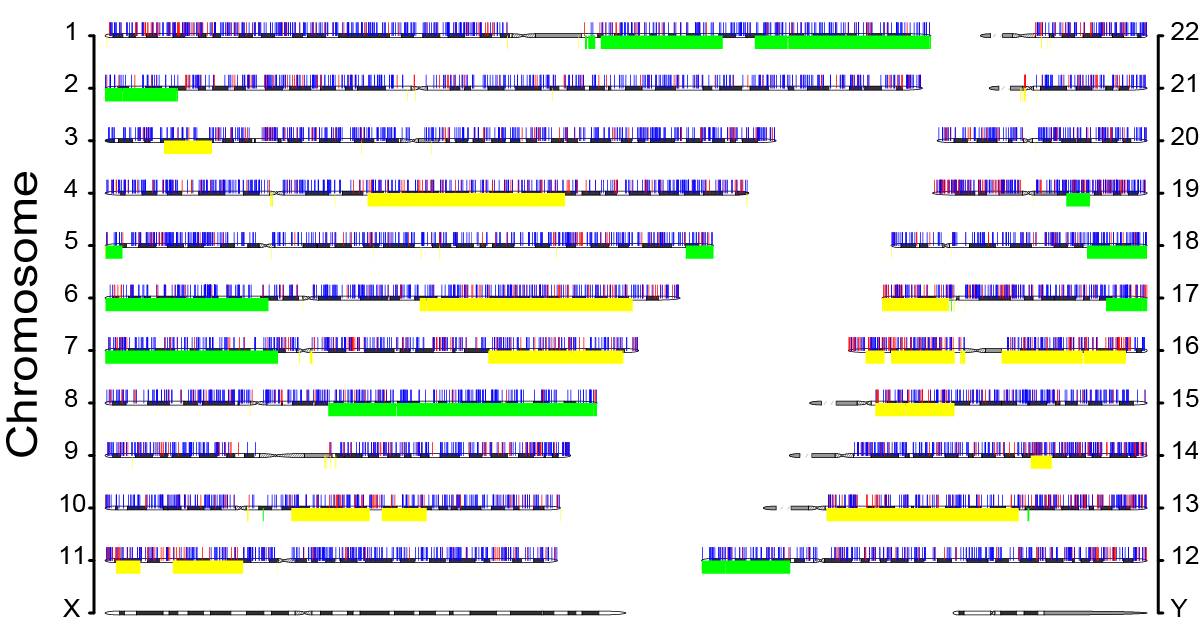

Supplement: Additional file 6: Figure S1. — Chromosomal distributions of LOHs, GOHs and CNVs in cancer samples. Symbols representing the different types of mutations are given in legend of Fig. 1. [file 12920_2015_104_MOESM6_ESM.zip › Figure S1/Figure S1H Liver (WGS).tif]
