# Supplementary material for: Massive interstitial copy-neutral loss-of-heterozygosity as evidence for cancer being a disease of the DNA-damage response
Source: BMC Med Genomics. 2015 Jul 25;8:42. doi: 10.1186/s12920-015-0104-2 (PMC4515014; doi:10.1186/s12920-015-0104-2)
Supplement: Additional file 10: Table S8. — Proteins identified by CIPHER as high-risk for the solid tumor group. The gene ranks indicated in % for the high-risk genes in the four different types of cancers were predicted by CIPHER. Covered length refers to the average length of gene sequence captured by the 30 AluScans analyzed in Table 2. [file 12920_2015_104_MOESM10_ESM.docx]

**Table S8. Proteins identified by CIPHER as high-risk for the solid tumor group. The gene ranks indicated in % for the high-risk genes in the four different types of cancers were predicted by CIPHER. Covered length refers to the average length of gene sequence captured by the 30 AluScans analyzed in Table 2.**

| **High-risk gene** | **Glioma** | **Lung**  **cancer** | **Liver**  **cancer** | **Gastric**  **cancer** | **Gene**  **length (bp)** | **Covered**  **length (bp)** | **% of**  **gene covered** |
| --- | --- | --- | --- | --- | --- | --- | --- |
| CDH1 |  |  |  | 0.17% | 98250 | 2340.2 | 2.38 |
| DLGAP1 | 3.86% |  |  | 0.08% | 959237 | 3755.1 | 0.39 |
| CTNNA3 |  |  |  | 6.7% | 1776225 | 6672.5 | 0.38 |
| RPS6KB1 |  |  | 1.57% | 2.32% | 57380 | 3570.8 | 6.22 |
| SAE1 | 2.25% | 1.18% |  |  | 79814 | 8860.6 | 11.10 |
| SP1 | 0.83% | 0.96% |  |  | 36248 | 1805.7 | 4.98 |
| DNAJA3 |  |  | 0.78% |  | 30970 | 4205.5 | 13.58 |
| AXIN1 |  |  | 2.13% |  | 65237 | 1182.0 | 1.81 |
| CHEK2 | 3.34% |  | 2.97% |  | 54092 | 2985.3 | 5.52 |
| TP53 | 0.81% | 3.46% | 5.84% | 4.27% | 19149 | 707.1 | 3.69 |
| APC | 0.66% |  | 2.23% | 0.35% | 108381 | 934.8 | 0.86 |
| GSK3B | 1.62% |  | 4.1% | 0.65% | 272463 | 771.2 | 0.28 |
| CTNNB1 | 3.39% |  | 0.78% | 0.41% | 40998 | 0.0 | 0.00 |
| MYC | 0.06% | 3.09% | 1.26% | 2.82% | 5366 | 0.0 | 0.00 |
| UBC | 1.73% |  |  |  | 3396 | 0.0 | 0.00 |
