# Supplementary material for: Massive interstitial copy-neutral loss-of-heterozygosity as evidence for cancer being a disease of the DNA-damage response
Source: BMC Med Genomics. 2015 Jul 25;8:42. doi: 10.1186/s12920-015-0104-2 (PMC4515014; doi:10.1186/s12920-015-0104-2)
Supplement: Supplementary file 12 [file 12920_2015_104_MOESM12_ESM.docx]

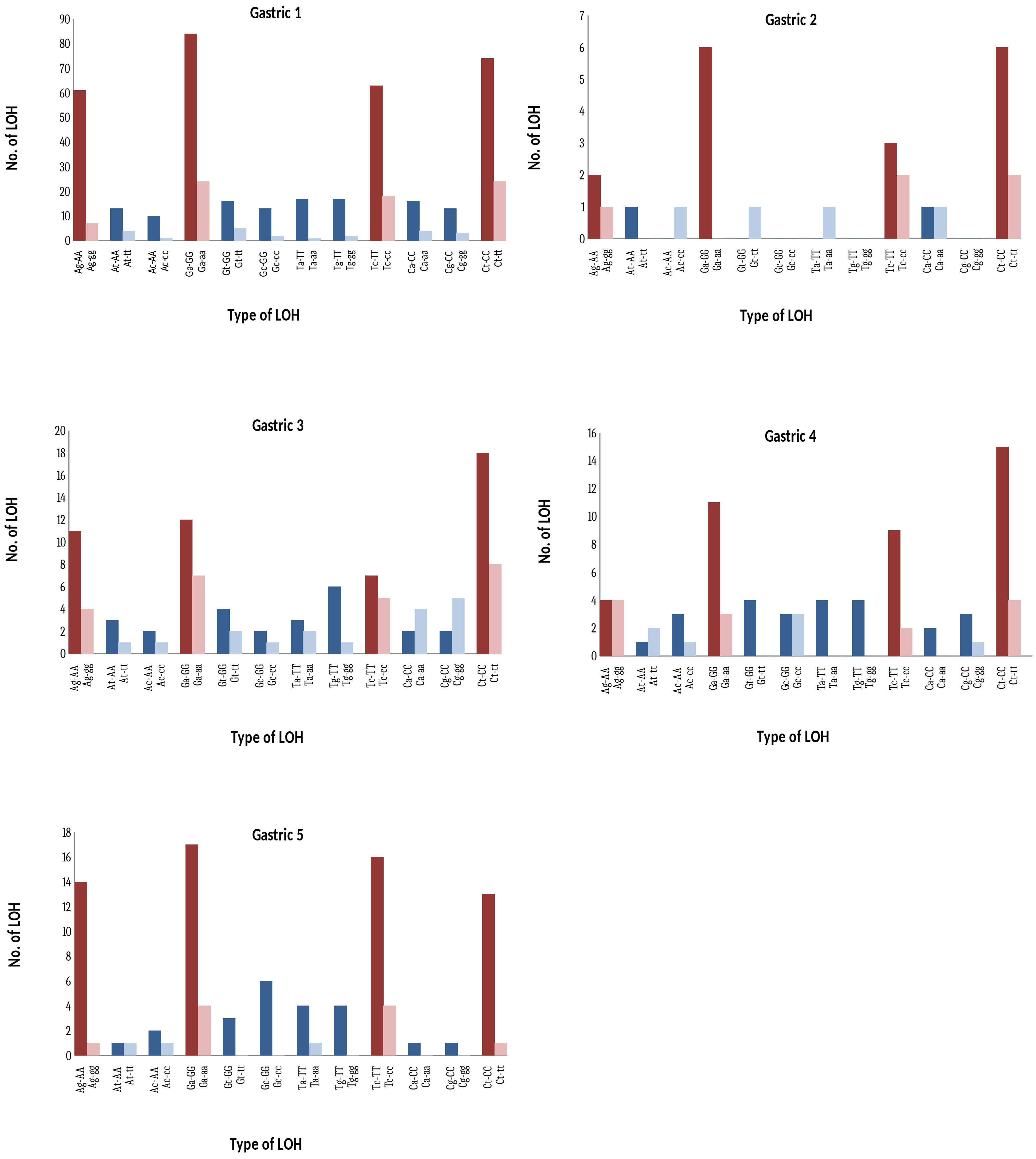
Gastric 1
No. of LOH
0
10
20
30
40
50
60
70
80
90
Ag-AA
Ag-gg
At-AA
At-tt
Ac-AA
Ac-cc
Ga-GG
Ga-aa
Gt-GG
Gt-tt
Gc-GG
Gc-cc
Ta-TT
Ta-aa
Tg-TT
Tg-gg
Tc-TT
Tc-cc
Ca-CC
Ca-aa
Cg-CC
Cg-gg
Ct-CC
Ct-tt
Type of LOH
Gastric 2
No. of LOH
0
1
2
3
4
5
6
7
Type of LOH
Gastric 3
No. of LOH
0
2
4
6
8
10
12
14
16
18
20
Type of LOH
Gastric 4
No. of LOH
0
2
4
6
8
10
12
14
16
Type of LOH
Gastric 5
No. of LOH
0
2
4
6
8
10
12
14
16
18
Type of LOH

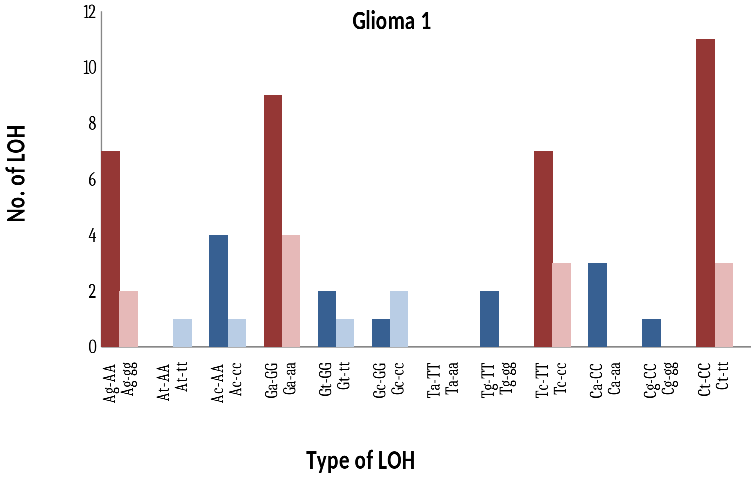
Glioma 1
No. of LOH
0
2
4
6
8
10
12
Ag-AA Ag-gg
At-AA At-tt
Ac-AA Ac-cc
Ga-GG Ga-aa
Gt-GG Gt-tt
Gc-GG Gc-cc
Ta-TT Ta-aa
Tg-TT Tg-gg
Tc-TT Tc-cc
Ca-CC Ca-aa
Cg-CC Cg-gg
Ct-CC Ct-tt
Type of LOH

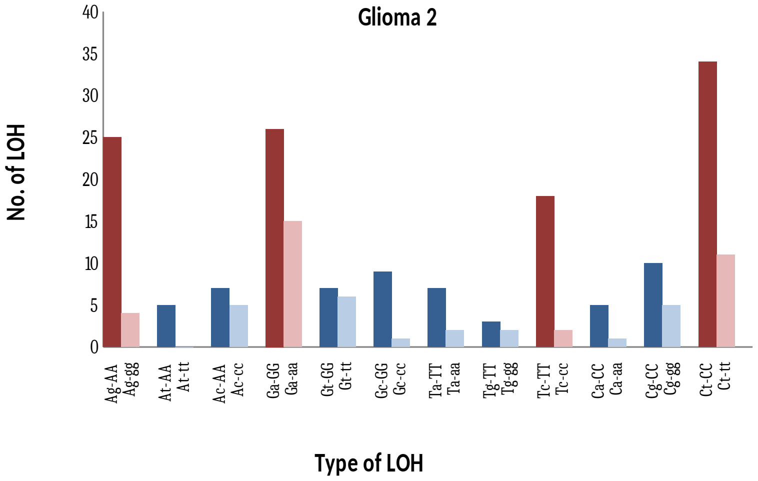
Glioma 2
No. of LOH
0
5
10
15
20
25
30
35
40
Ag-AA Ag-gg
At-AA At-tt
Ac-AA Ac-cc
Ga-GG Ga-aa
Gt-GG Gt-tt
Gc-GG Gc-cc
Ta-TT Ta-aa
Tg-TT Tg-gg
Tc-TT Tc-cc
Ca-CC Ca-aa
Cg-CC Cg-gg
Ct-CC Ct-tt
Type of LOH

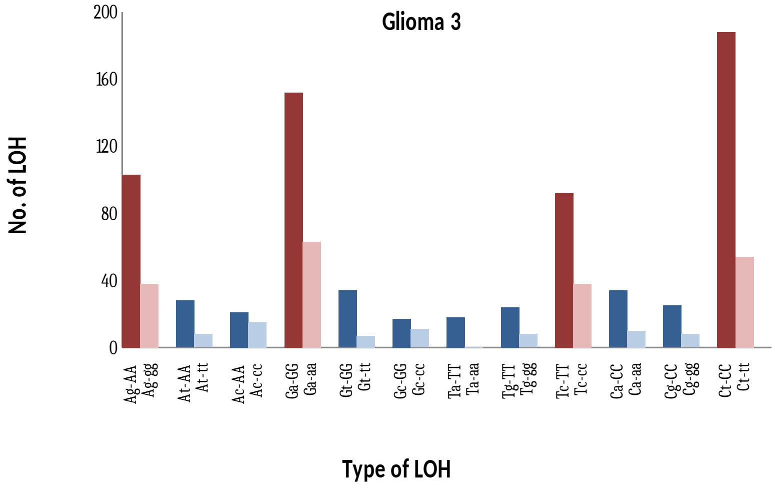
Glioma 3
No. of LOH
0
40
80
120
160
200
Ag-AA Ag-gg
At-AA At-tt
Ac-AA Ac-cc
Ga-GG Ga-aa
Gt-GG Gt-tt
Gc-GG Gc-cc
Ta-TT Ta-aa
Tg-TT Tg-gg
Tc-TT Tc-cc
Ca-CC Ca-aa
Cg-CC Cg-gg
Ct-CC Ct-tt
Type of LOH

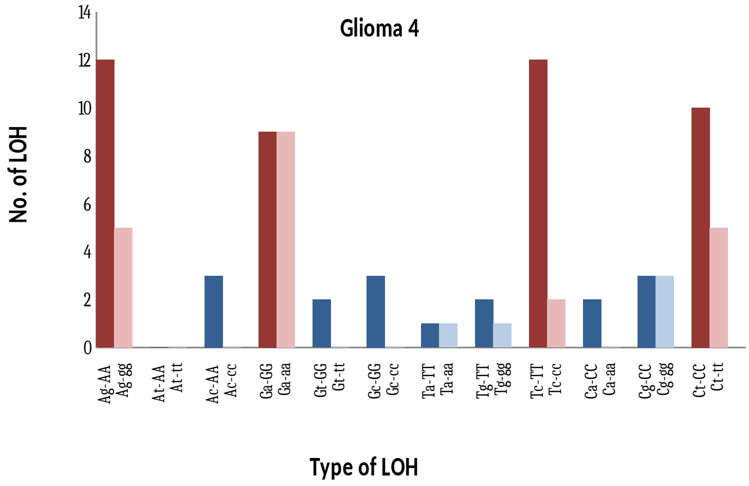
Glioma 4
No. of LOH
0
2
4
6
8
10
12
14
Ag-AA Ag-gg
At-AA At-tt
Ac-AA Ac-cc
Ga-GG Ga-aa
Gt-GG Gt-tt
Gc-GG Gc-cc
Ta-TT Ta-aa
Tg-TT Tg-gg
Tc-TT Tc-cc
Ca-CC Ca-aa
Cg-CC Cg-gg
Ct-CC Ct-tt
Type of LOH

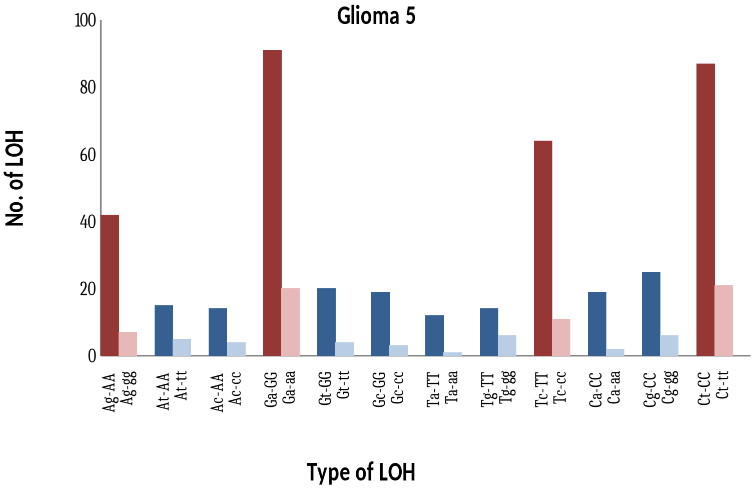
Glioma 5
No. of LOH
0
20
40
60
80
100
Ag-AA Ag-gg
At-AA At-tt
Ac-AA Ac-cc
Ga-GG Ga-aa
Gt-GG Gt-tt
Gc-GG Gc-cc
Ta-TT Ta-aa
Tg-TT Tg-gg
Tc-TT Tc-cc
Ca-CC Ca-aa
Cg-CC Cg-gg
Ct-CC Ct-tt
Type of LOH

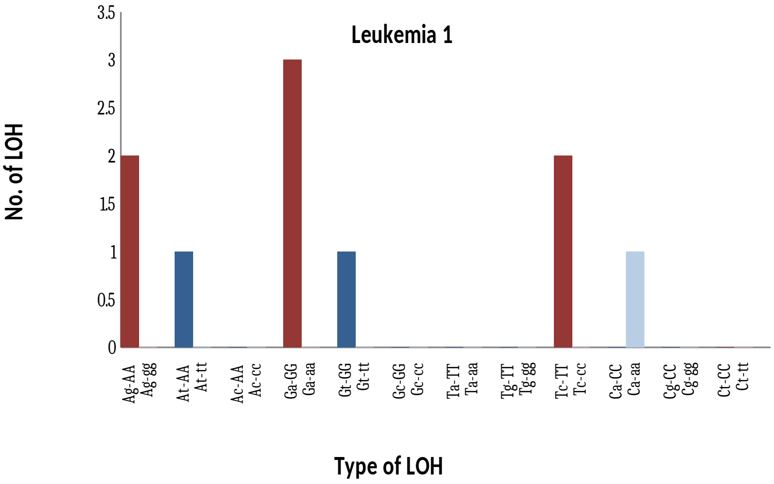
Leukemia 1
No. of LOH
3.5
3
2.5
2
1.5
1
0.5
0
Ag-AA Ag-gg At-AA At-tt Ac-AA Ac-cc Ga-GG Ga-aa Gt-GG Gt-tt Gc-GG Gc-cc Ta-TT Ta-aa Tg-TT Tg-gg Tc-TT Tc-cc Ca-CC Ca-aa Cg-CC Cg-gg Ct-CC Ct-tt
Type of LOH

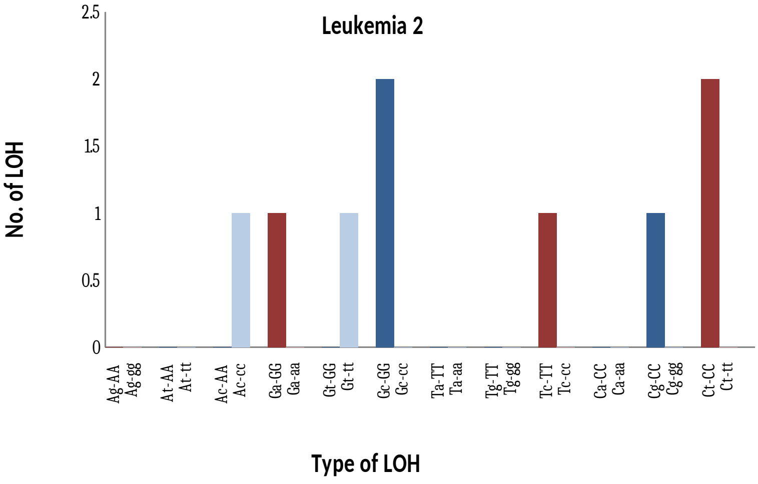
Leukemia 2
No. of LOH
2.5
2
1.5
1
0.5
0
Ag-AA Ag-gg At-AA At-tt Ac-AA Ac-cc Ga-GG Ga-aa Gt-GG Gt-tt Gc-GG Gc-cc Ta-TT Ta-aa Tg-TT Tg-gg Tc-TT Tc-cc Ca-CC Ca-aa Cg-CC Cg-gg Ct-CC Ct-tt
Type of LOH

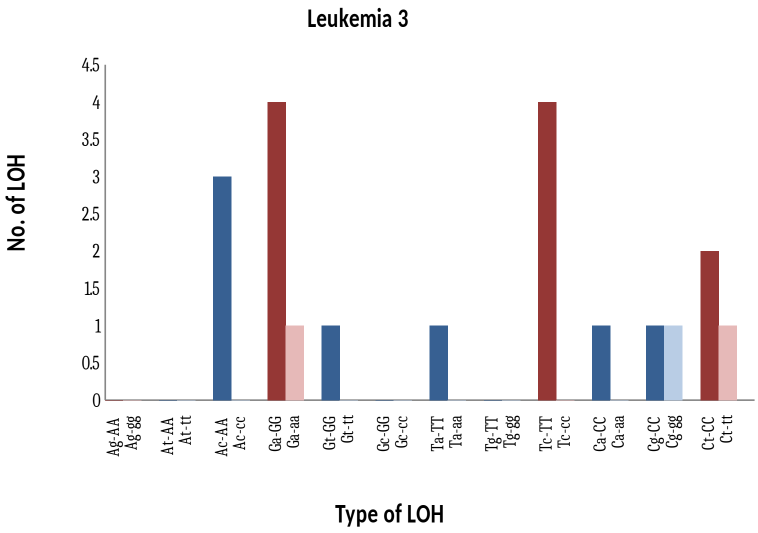
Leukemia 3
No. of LOH
4.5
4
3.5
3
2.5
2
1.5
1
0.5
0
Ag-AA Ag-gg At-AA At-tt Ac-AA Ac-cc Ga-GG Ga-aa Gt-GG Gt-tt Gc-GG Gc-cc Ta-TT Ta-aa Tg-TT Tg-gg Tc-TT Tc-cc Ca-CC Ca-aa Cg-CC Cg-gg Ct-CC Ct-tt
Type of LOH

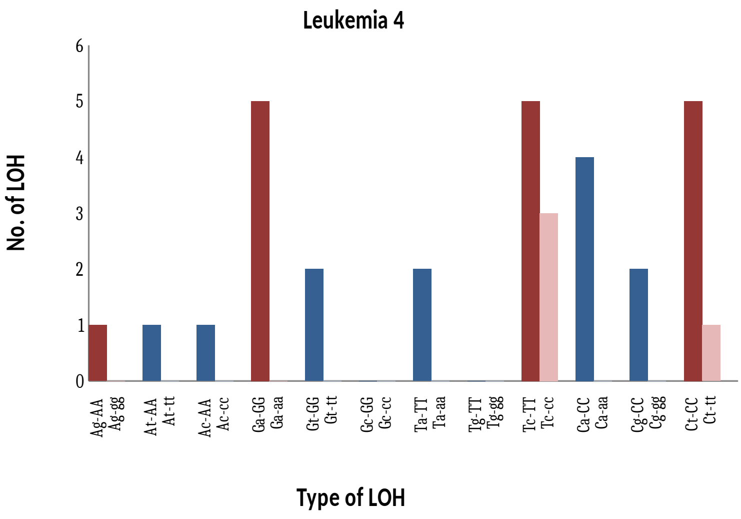
Leukemia 4
No. of LOH
6
5
4
3
2
1
0
Ag-AA Ag-gg At-AA At-tt Ac-AA Ac-cc Ga-GG Ga-aa Gt-GG Gt-tt Gc-GG Gc-cc Ta-TT Ta-aa Tg-TT Tg-gg Tc-TT Tc-cc Ca-CC Ca-aa Cg-CC Cg-gg Ct-CC Ct-tt
Type of LOH

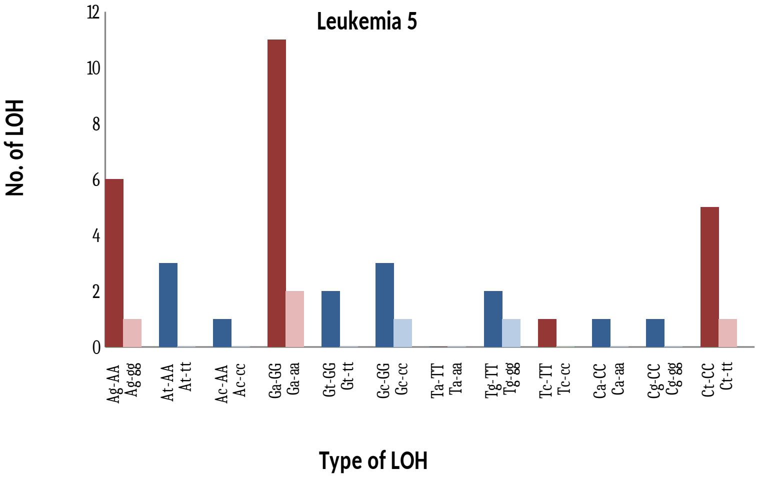
Leukemia 5
No. of LOH
12
10
8
6
4
2
0
Ag-AA Ag-gg At-AA At-tt Ac-AA Ac-cc Ga-GG Ga-aa Gt-GG Gt-tt Gc-GG Gc-cc Ta-TT Ta-aa Tg-TT Tg-gg Tc-TT Tc-cc Ca-CC Ca-aa Cg-CC Cg-gg Ct-CC Ct-tt
Type of LOH

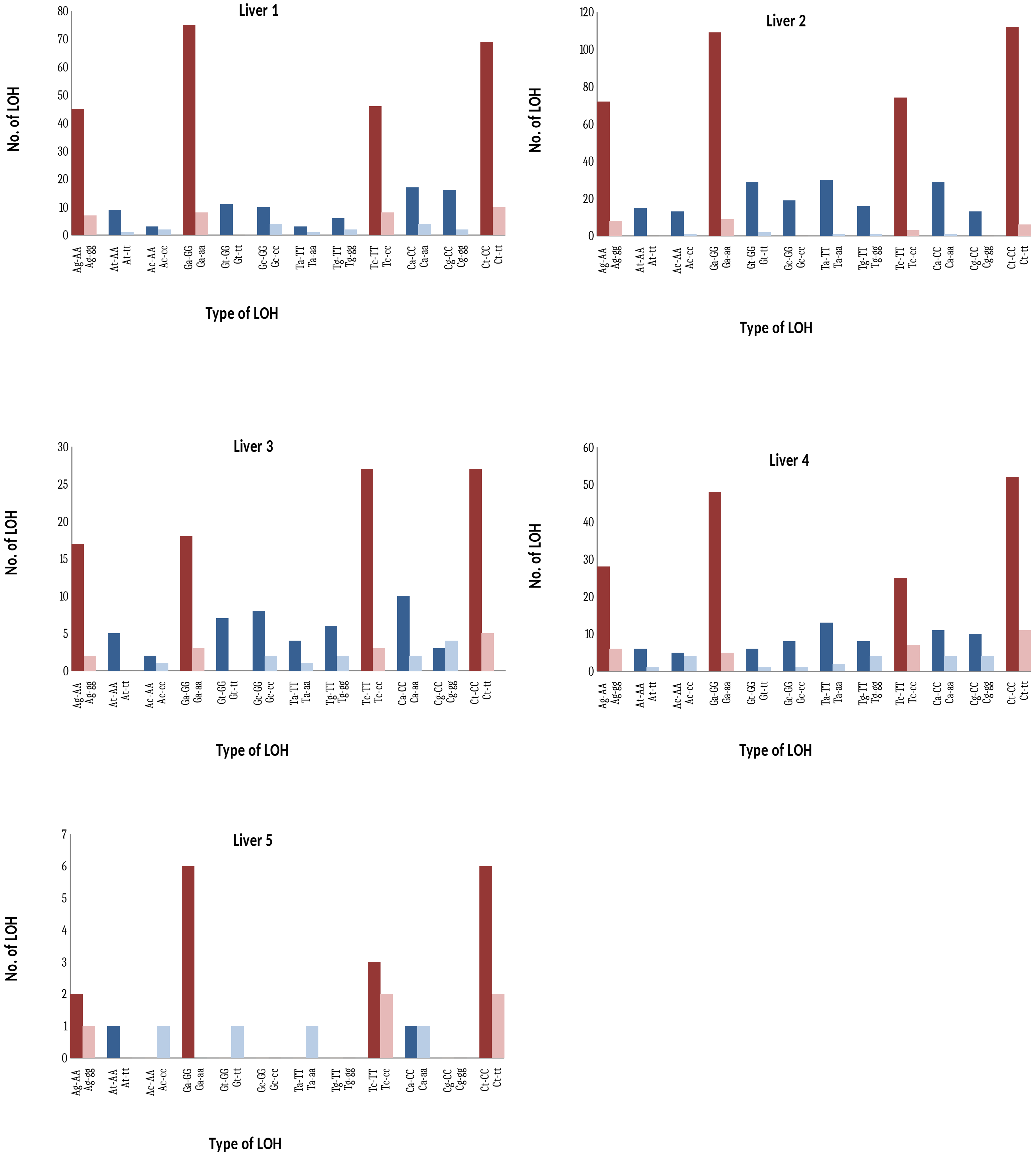
Liver 1
No. of LOH
Type of LOH
Liver 2
No. of LOH
Type of LOH
Liver 3
No. of LOH
Type of LOH
Liver 4
No. of LOH
Type of LOH
Liver 5
No. of LOH
Type of LOH
Ag-AA Ag-gg At-AA At-tt Ac-AA Ac-cc Ga-GG Ga-aa Gt-GG Gt-tt Gc-GG Gc-cc Ta-TT Ta-aa Tg-TT Tg-gg Tc-TT Tc-cc Ca-CC Ca-aa Cg-CC Cg-gg Ct-CC Ct-tt

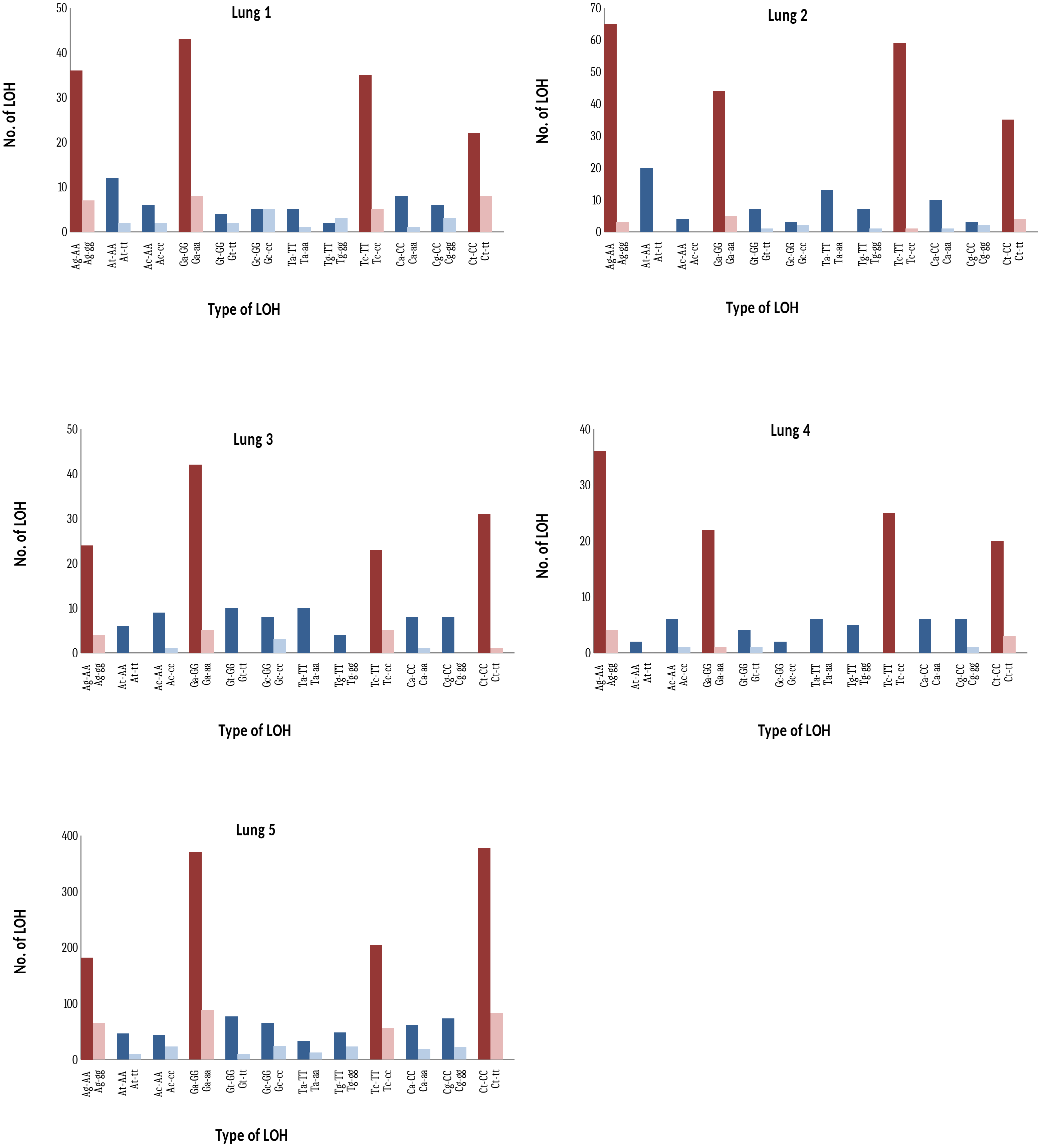
Lung 1
No. of LOH
0
10
20
30
40
50
Ag-AA
Ag-gg
At-AA
At-tt
Ac-AA
Ac-cc
Ga-GG
Ga-aa
Gt-GG
Gt-tt
Gc-GG
Gc-cc
Ta-TT
Ta-aa
Tg-TT
Tg-gg
Tc-TT
Tc-cc
Ca-CC
Ca-aa
Cg-CC
Cg-gg
Ct-CC
Ct-tt
Type of LOH
Lung 2
No. of LOH
0
10
20
30
40
50
60
70
Type of LOH
Lung 3
No. of LOH
0
10
20
30
40
50
Type of LOH
Lung 4
No. of LOH
0
10
20
30
40
Type of LOH
Lung 5
No. of LOH
0
100
200
300
400
Type of LOH

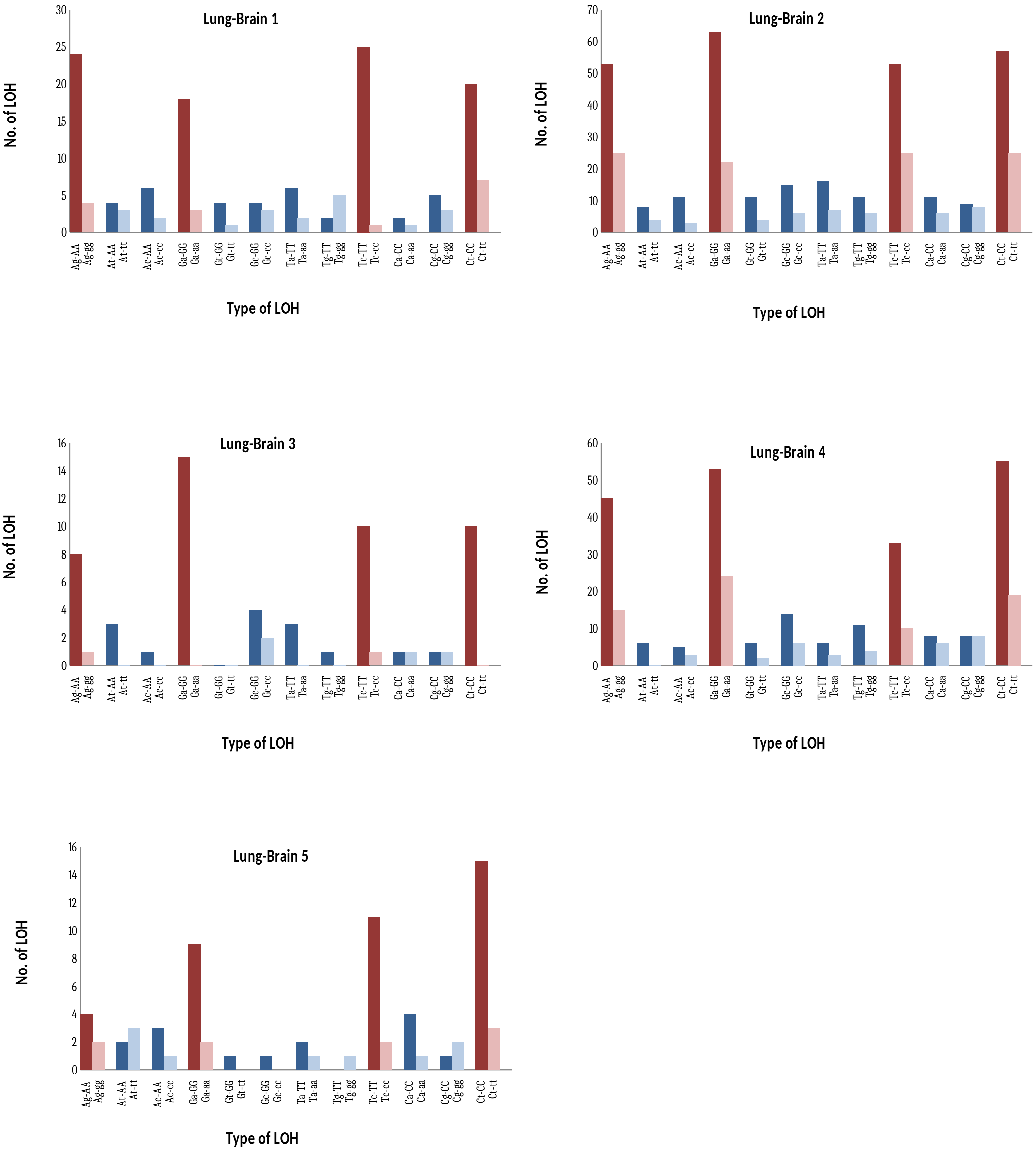
Lung-Brain 1
No. of LOH
Type of LOH
Lung-Brain 2
No. of LOH
Type of LOH
Lung-Brain 3
No. of LOH
Type of LOH
Lung-Brain 4
No. of LOH
Type of LOH
Lung-Brain 5
No. of LOH
Type of LOH
Ag-AA Ag-gg At-AA At-tt Ac-AA Ac-cc Ga-GG Ga-aa Gt-GG Gt-tt Gc-GG Gc-cc Ta-TT Ta-aa Tg-TT Tg-gg Tc-TT Tc-cc Ca-CC Ca-aa Cg-CC Cg-gg Ct-CC Ct-tt
